# Neural Control and Online Learning for Speed Adaptation of Unmanned Aerial Vehicles

**DOI:** 10.3389/fncir.2022.839361

**Published:** 2022-04-25

**Authors:** Vatsanai Jaiton, Kongkiat Rothomphiwat, Emad Ebeid, Poramate Manoonpong

**Affiliations:** ^1^Bio-Inspired Robotics and Neural Engineering Laboratory, School of Information Science and Technology, Vidyasirimedhi Institute of Science and Technology, Rayong, Thailand; ^2^SDU UAS Centre (Unmanned Aerial Systems), The Mærsk Mc-Kinney Møller Institute, University of Southern Denmark, Odense, Denmark; ^3^Embodied AI and Neurorobotics Laboratory, SDU Biorobotics, The Mærsk Mc-Kinney Møller Institute, University of Southern Denmark, Odense, Denmark

**Keywords:** drone, adaptive neural control, neural proactive control, online learning, speed adaptation, obstacle avoidance

## Abstract

Unmanned aerial vehicles (UAVs) are involved in critical tasks such as inspection and exploration. Thus, they have to perform several intelligent functions. Various control approaches have been proposed to implement these functions. Most classical UAV control approaches, such as model predictive control, require a dynamic model to determine the optimal control parameters. Other control approaches use machine learning techniques that require multiple learning trials to obtain the proper control parameters. All these approaches are computationally expensive. Our goal is to develop an efficient control system for UAVs that does not require a dynamic model and allows them to learn control parameters online with only a few trials and inexpensive computations. To achieve this, we developed a neural control method with fast online learning. Neural control is based on a three-neuron network, whereas the online learning algorithm is derived from a neural correlation-based learning principle with predictive and reflexive sensory information. This neural control technique is used here for the speed adaptation of the UAV. The control technique relies on a simple input signal from a compact optical distance measurement sensor that can be converted into predictive and reflexive sensory information for the learning algorithm. Such speed adaptation is a fundamental function that can be used as part of other complex control functions, such as obstacle avoidance. The proposed technique was implemented on a real UAV system. Consequently, the UAV can quickly learn within 3–4 trials to proactively adapt its flying speed to brake at a safe distance from the obstacle or target in the horizontal and vertical planes. This speed adaptation is also robust against wind perturbation. We also demonstrated a combination of speed adaptation and obstacle avoidance for UAV navigations, which is an important intelligent function toward inspection and exploration.

## 1. Introduction

**Figure 1:**
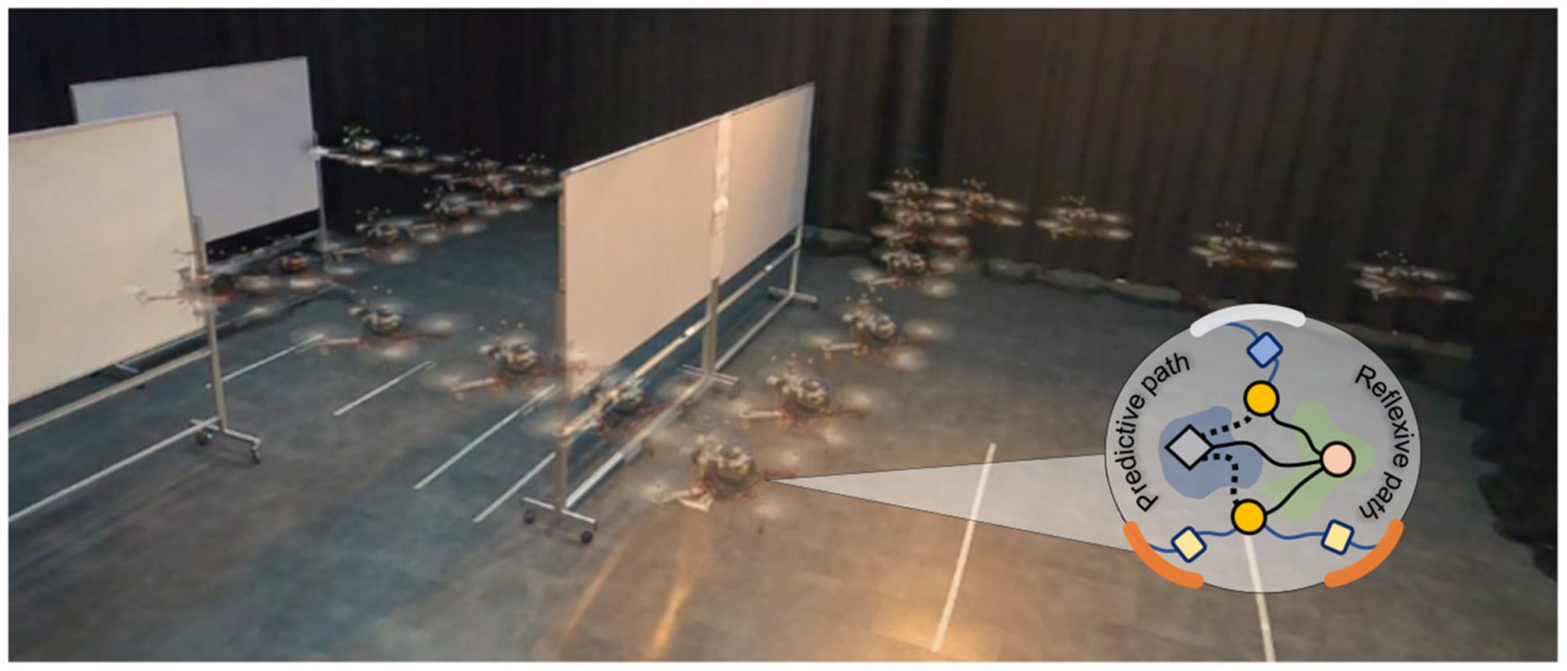
Speed adaptation algorithm, based on neural control with online correlation-based learning capability (or considered as neural proactive control, insert circle). A reflexive path is the path for generating reactive behavior, while a predictive path is the path for performing online learning and generating adaptive behavior. The algorithm serves as a primitive control function for obstacle avoidance. It allows the UAV to automatically fly (see from right to left) and proactively adapt its flying speed and brake at a safe distance to avoid an obstacle.

Unmanned aerial vehicles (UAVs) or drones are used in various challenging applications, such as industrial inspection (Kocerab et al., 2019; Ribeiro et al., 2019; Yao et al., 2021), exploration and surveying (Dai et al., 2019; Jiao et al., 2020), and rescue and emergency response (Karaca et al., 2018; Deng et al., 2019), all of which require the UAV to perform a given mission automatically. Thus, to effectively complete the mission, UAVs should have many intelligent functions, such as obstacle avoidance (Figure 1), exploration, searching, navigation, object transportation, and autonomous landing. However, a basic safety function is crucial. In particular, when performing missions in complex environments, such as caves, tunnels, indoor areas, or urban areas with many obstacles, the UAV should be in a safe condition before performing other advanced functions. Therefore, both basic safety and advanced functions should be considered in the development of fully autonomous UAVs to handle unknown environments. To achieve this, different UAV control techniques have been developed based on two main principles: model-based and model-free controls.

Model-based control requires kinematic and system dynamic models to control UAV behavior. Widely used control methods, which rely on models, include feedback proportional–integral–derivative (PID) control and model predictive control (MPC). For PID control, the control parameters are tuned to match the models to obtain the desired behaviors (Sangyam et al., 2010; Goodarzi et al., 2013). It is mostly used for basic functions such as attitude and position control, while the MPC is used to optimize the control parameters by predicting the future state of the UAV. The MPC is deployed with both basic functions (e.g., precision Dentler et al., 2016; Feng et al., 2018; Jardine et al., 2019, environmental disturbance compensation Chikasha and Dube, 2017), and advanced functions (e.g., obstacle avoidance and navigation, Bareiss et al., 2017; Yang et al., 2019; Dai et al., 2020). In addition, it is used in trajectory tracking to control the UAV, ensuring that it follows the planned path (Baca et al., 2018; Tordesillas et al., 2019; Invernizzi et al., 2020). However, in advanced functions, the control system needs to perceive the frontal (Tordesillas et al., 2019) or surrounding (Bareiss et al., 2017) environmental information of the UAV to plan a safe flying trajectory. Therefore, complex sensors (e.g., cameras and laser scanners) have to be installed. Furthermore, their complex signal processing to predict the future state and perceive environmental information requires high computational effort, particularly in complex environments.

Model-free control, however, does not require a system dynamic model. This technique includes reactive and learning-based control for controlling UAVs to perform complex missions. For reactive-based control (Rambabu et al., 2015; Cheong et al., 2016; Ki et al., 2018; Huang et al., 2019), sensory (feedback) signals are used to generate reactive control commands to the UAV, such as stop and avoidance commands when obstacles are detected. Reactive-based control typically requires less computational effort than other methods, such as the MPC. However, the system design of this technique should be considered, including sensor installation, sensor detection range, and parameter tuning, to ensure the effectiveness of the control system. For automatic parameter tuning or optimization, various machine learning methods have been applied including deep learning (Palossi et al., 2019, 2022; Varshney et al., 2019), reinforcement learning (RL) (Shin et al., 2019; Wang et al., 2019; Lin et al., 2020), and an evolutionary algorithm (EA) (Fu et al., 2018; Yazid et al., 2019). Although they have become more popular in recent years, their learning processes are typically time-consuming and computationally expensive, requiring a large amount of data and multiple learning trials or iterations. Thus, an offline learning process with prepared data or a simulation is used before transferring the learned control parameters to a real UAV. Therefore, it is important to ensure that all possible situations are introduced during the learning process; otherwise, the UAV may fail to address unexpected situations (i.e., situations that have not been trained or learned before).

Based on the aforementioned problems, we propose a neural proactive control, derived from a neural control network and correlation-based online learning. The proposed method does not require a dynamic model, multiple learning trials, or even a large amount of data and processing power to determine the optimal control parameters. In addition, it can be directly implemented on a real UAV to learn online in real time. The neural control method serves as a model-free proactive control using a learning technique to proactively provide system adaptability to interact with the environment. Here, we apply this control method to the speed adaptation control of a UAV, allowing it to proactively adapt its flying speed and stop at a safe distance from the obstacle or target in horizontal and vertical planes. This speed adaptation is a primitive or fundamental behavior for building more complex behaviors, such as approaching/goal-directed and obstacle avoidance. All these complex behaviors require primitive behavior that can adapt or change the UAV's flying speed to stop in front of its target before performing the next behavior. Speed adaptation control relies on only one input signal from a compact optical distance measurement sensor. The proposed algorithm was implemented on a real UAV and evaluated in a real environment. The aim is to automatically determine the optimal control parameters through a learning mechanism that learns from the actual system dynamics without system and environmental models. The UAV can learn the speed control parameters (gains) online to proactively adapt its flying speed and brake at a proper rate for various maximum speeds in the horizontal and vertical planes (i.e., flying speed adaptation in the x, y, or z direction). This learning process is data efficient and can be completed in 3–4 trials. Furthermore, the control system is robust against wind perturbation.

We additionally evaluated the online learning experiment of the proposed neural proactive control on a simulated UAV platform (Gazebo) to show that our control technique can be easily applied to another UAV platform without a dynamic model or a specific parameter setup for the UAV. Thus, the proposed neural control can be considered as a general fast online learning system for addressing the speed adaptation of different UAVs with different maximum flying speeds. The performance of our neural proactive control was compared with that of the MPC (Varshney et al., 2019; Lindqvist et al., 2020; Wang et al., 2021). The results show that both methods can effectively control the UAV to adapt its flying speed at an appropriate rate for various maximum speeds. However, there are more system requirements for implementing the MPC. For example, the MPC requires a specific dynamic model of the UAV and is computationally expensive, while the neural control does not require any model and is computationally inexpensive (i.e., 56.32 and 99.47% less computational time for one iteration and total learning/optimization, respectively; see Table 1 in Section 3). Finally, we presented a use case of speed adaptation control as a primitive adaptive function of obstacle avoidance in a real environment with static and dynamic obstacles, which enables the UAV to fly effectively and become closer to the obstacle before avoiding it, which is significant for applications such as inspection and exploration.

**Table 1:** Comparison table showing the difference between implementing the NPC and the explicit MPC as the main controller of the simulated UAV speed adaptation control.

**Comparison Item**	**Control method**	
	**NPC**	**Explicit MPC**
Dynamic model requirement	✗	✓
Learning and optimization requirement	✓(online)	✓(offline)
Computational time for generating a control command (average)	0.38 ms (56.32%)	0.87 ms
Total learning and optimization time (average)	464.81 s (99.47%)	24.48 h

*The percentage values in the table represent that the NPC requires less computational times than the explicit MPC: 56.32% for one iteration and 99.47% for total learning/optimization*.

Taken together, this study has the following main contributions:

- General and robust control with fast online learning and data efficiency for UAV speed adaptation. It can handle different maximum flying speeds in the horizontal and vertical planes and unexpected wind perturbation. The online learning method is computationally inexpensive and does not require multiple trials and a large data collection to automatically optimize the speed control parameter(s). Consequently, it is more efficient than traditional model predictive control and machine learning-based control.
- Demonstrations for real-world speed adaptation and obstacle avoidance of a UAV in a variety of situations, including encountering static and dynamic obstacles, flying under unexpected wind perturbation, and flying in different directions (i.e., x, y, and z directions).
- Available source code of the neural control method in our Git repository^1^ to allow replication.

The remainder of this article is organized as follows. In Section 2, we present an overview of the proposed control system and details of the speed adaptation algorithm based on neural proactive control (neural control and online learning). Section 3 provides a description of the UAV system used as our experimental platform and the experiments and results of the proactive control technique at different flying speeds in real and simulated environments. We also present a performance comparison of the proposed control technique with classical reactive control and MPC techniques, and a use case for obstacle avoidance of the proposed control technique. Finally, Sections 4 and 5 provide the discussion and conclusions, respectively.

## 2. Materials and Methods

**Figure 2:**
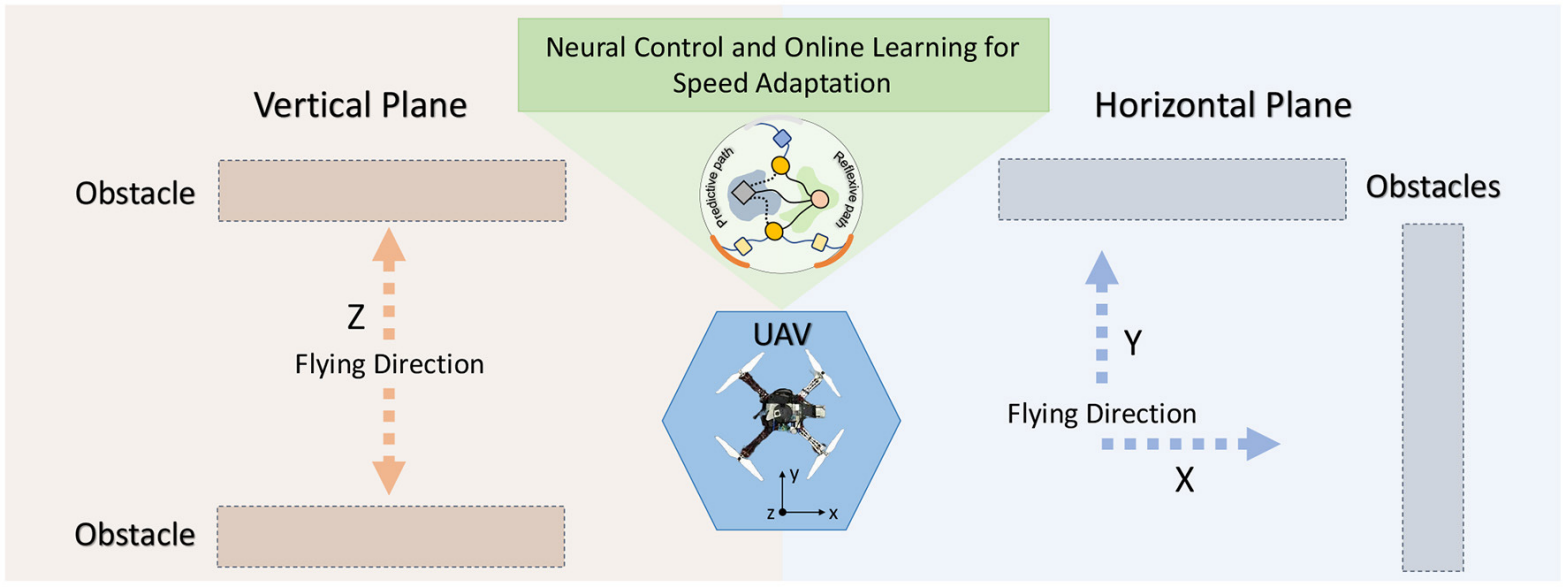
Setup for testing and demonstrating the performance of the control system. In this scenario, a UAV flies at a given speed toward an obstacle. When it becomes close to the obstacle, the learning system allows it learn to gradually adapt its speed and brake at a safe distance from the obstacle. The proposed control and learning system is not restricted to a certain flying direction. It can be used to adapt the flying speed of a UAV in the horizontal and vertical planes (i.e., x, y, or z direction).

In this section, we describe the neural control and online learning system used for speed adaptation and obstacle avoidance (Figure 2). First, we provide an overview of the control system, including its requirements and potential applications. Subsequently, we describe the principle and functionality of neural control and online learning system. Finally, we demonstrate how we use the output of the control system for the behavioral control of UAVs.

### 2.1. Control System Overview

**Figure 3:**
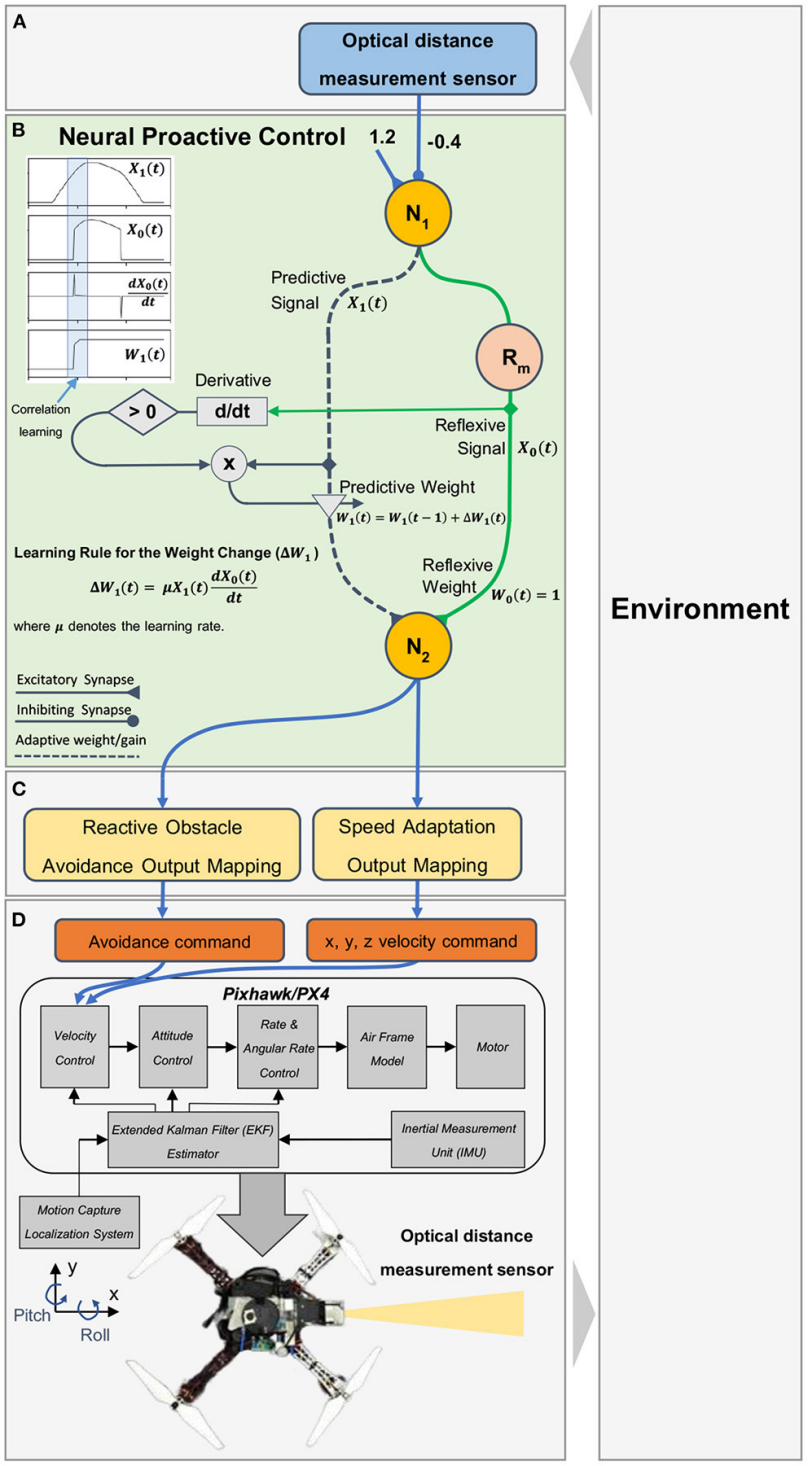
Neural proactive closed-loop controller for speed adaptation and obstacle avoidance. The controller consists of four main modules: **(A)** the input module, using an optical distance sensor to detect the distance between a UAV and frontal obstacle; **(B)** the neural control module with the ICO learning mechanism as the main component of the proactive controller (the dark blue dashed line indicating the predictive path and the green line indicating the reflexive path), consisting of three neurons (two discrete-time non-spiking neurons (N_1_ and N_2_; see Equation 1) and one modified ReLU neuron (R_*m*_; see Equation 2); **(C)** the output mapping module that maps the neural output to the low-level control by separating the output N_2_ into speed adaptation control and obstacle avoidance control; **(D)** the flight controller (Pixhawk/PX4) for low-level control (i.e., velocity and attitude control). Note that the inset graph in **(B)** illustrates the predictive *X*_1_ and reflexive *X*_0_ signals, their correlation (*X*_1_*dX_0_*/dt)*, and weight adaptation *W*_1_. In our implementation here, only the positive change of the reflexive signal is used for weight adaptation.

The control system is based on a modular structure (Figure 3). It consists of an input module with distance measurement sensor feedback, a neural control and online learning module, an output mapping module, and a low-level flight control module. In this study, the neural control and online learning module is the main component developed and applied to enable a UAV to adaptively reduce its speed (or determine the appropriate rate of speed adaptation) to brake before colliding with an obstacle (Figure 2) in the horizontal and vertical planes. The rate of speed adaptation is proportional to the plastic synaptic weight of the neural control system. An input correlation (ICO) learning mechanism was employed to learn the synaptic weight (see Section 2.2). Our use case, described later, shows that a UAV can fly in an area with obstacles while gradually and continuously learning to determine a proper synaptic weight for speed adaptation at any given flying speed. This neural control and online learning system requires only obstacle detection information at different ranges (here, a simple distance measurement sensor; Figure 3) to generate an adaptive speed control output (see Section 2.3) without system and environmental models. Thus, in principle, it can be utilized for speed adaptation in other mobile robot systems, where its speed control output should be mapped to the final low-level speed control command of the corresponding robot system.

### 2.2. Neural Control and Online Learning for Speed Adaptation

To achieve speed adaptation of the UAVs, we utilized neural control with the ICO learning mechanism^2^ as a proactive neurocomputing system to generate an adaptive control signal to the UAV. Neural control is based on a three-neuron network, as shown in Figure 3B. Neurons N_1_ and N_2_ of the network were modeled as discrete-time non-spiking neurons. The activity, *a*_*i*_, of each neuron was developed based on the following equation:


(1) $$\begin{array}{l} a_{i}\left(t\right)=\overset{n}{\underset{j=1}{\sum }}W_{ij}\cdot o_{j}\left(t-1\right)+B_{i}\;i=1,\ldots ,n, \end{array}$$

where *n* denotes the number of neurons, *B*_*i*_ is an internal bias term or a sensory input to neuron *i*, and *W*_*ij*_ represents the synaptic weight of the connection from neuron *j* to neuron *i*. The output *o*_*i*_ of the neuron is calculated using a linear transfer function (*o*_*i*_(*t*) = *a*_*i*_(*t*)).

Neuron R_*m*_ of the network was modeled as a modified rectifier neuron or modified ReLU. The activation function is defined as follows:


(2) $$\begin{array}{l} R_{m}(t)=\{\begin{array}{ll} o_{1}(t) & o_{1}(t)>1,\\ 0 & o_{1}(t)\leq 1, \end{array} \end{array}$$

where *o*_1_(*t*) is the output of N_1_ (Figure 3).

ICO learning is an unsupervised learning technique that uses a correlation-based learning rule between two input signals, mapped to different obstacle detection ranges. The first signal was mapped to long-range detection for prior occurrence and is called the “predictive signal” (*X*_1_). The second signal was mapped to short-range detection for later occurrence and is called the “reflexive signal” (*X*_0_). Both input signals were transmitted to the learner neuron (N_2_) through the predictive or plastic synaptic weight (*W*_1_) and reflexive weight (*W*_0_), respectively (Figure 3). The predictive weight can be learned or adapted based on the ICO learning rule using the correlation between the two input signals. The correlation was realized by multiplying the predictive signal and the positive change (derivative) of the reflexive signal. A learning rate (μ) was applied to scale the correlation and define the speed of the learning process. In this learning rule, only the predictive weight was allowed to change; thus, the reflexive weight was set to a fixed positive of 1.0. In our approach (Figure 3), the predictive signal was the output of N_1_, which uses a single input signal^3^ from the distance sensor, while the reflexive signal was the output of the modified rectifier neuron R_*m*_ (modified ReLU), which uses the N_1_ output as its input. The sensor was installed on the UAV to detect obstacles. The sensor signal was applied as the input signal of N_1_. It was mapped to a linear range by N_1_ using an input gain and bias of −0.4 and 1.2, respectively. The negative input gain basically inverts and scales the sensor signal, while the bias term offsets it, so the linear output of N_1_ can vary between 0.0 (i.e., the distance between the sensor and an obstacle is greater than 3.0 m) and 1.1 (i.e., the distance between the sensor and an obstacle is equal to 0.25 m)^4^. The output of N_1_ was separated into two paths: the predictive signal path that transmitted the input *X*_1_ to N_2_, and the reflexive signal path that transmitted the input *X*_0_ to N_2_. Here, the reflexive signal, which is based on the R_*m*_ output, is equal to the predictive signal when the predictive signal is higher than one and zero when the predictive signal is equal to or less than one, as described in Equation (2).

In this setup, the predictive weight was initially set to zero, and the reflexive weight was set to one. Based on the learning rule and initialized weight setting in the first trial, the output of N_2_ [i.e., *o*_2_*(t)* = *W*_0_*X*_0_*(t)* + *W*_1_*(t)X*_1_*(t)*] was activated once the reflexive signal occurred. In this situation, the UAV was controlled only by the reflexive signal. Consequently, the UAV braked immediately to avoid hitting the obstacle. However, if the flying speed is quite high, the UAV may collide with the obstacle. Meanwhile, the predictive weight was gradually updated during the occurrence of the reflexive signal through the correlation between these two signals, as described in Equation (3):


(3) $$\begin{array}{l} \Delta W_{1}\left(t\right)=\mu \frac{dX_{0}\left(t\right)}{dt}X_{1}\left(t\right), \end{array}$$

where Δ*W*_1_(*t*) denotes the change in the predictive weight. μ is the learning rate, which defines the speed at which the system can learn. To accelerate the learning process, we set the learning rate to 0.3. Appendix A provides more details on how different learning rates impact the system's performance.

The inset graph in Figure 3B shows the predictive and reflexive signals and their correlation for the predictive weight adaptation (*W*_1_). The predictive signal occurs earlier, whereas the reflexive signal in this setup occurs when the predictive signal is greater than a threshold (i.e., 1.0). The predictive weight is learned at the overlapping period between these two signals (when they correlate in time) based on the learning rule in Equation (3). Under this learning rule, we use only a correlation of a positive change of the reflexive signal (or a positive derivative) and the predictive signal to incrementally update the predictive weight until the reflexive signal is no longer present. This allows the UAV to proactively adapt or decrease its flying speed to stop in front of the obstacle in time. Algorithm 1 shows the pseudocode of the neural control and online learning, together with the learning process to determine the optimal predictive weight. Note that the predictive and reflexive weights can be considered as adaptive and fixed gains, respectively, for the speed adaptation control of the UAV.

**Algorithm 1:**
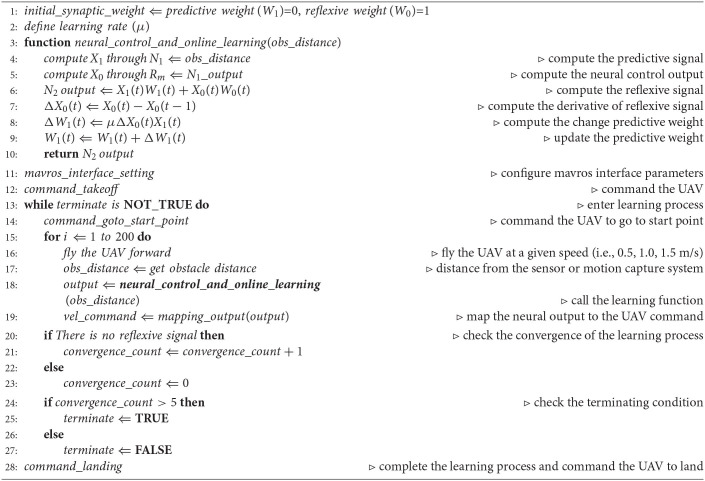
Neural Control and Online Learning.

Here, we performed the experiment and learning process in a real environment, allowing the control system to learn with the real dynamics of the UAV. At the beginning of the learning process, the UAV started to reduce its flying speed and braked when the reflexive signal occurred. The learning process was run continuously until the predictive weight became sufficiently strong. Subsequently, the UAV was controlled only by the predictive signal with an appropriate magnitude through the weight. Basically, the UAV was able to proactively decrease its speed and brake without the reflexive signal (i.e., decreasing its speed and breaking earlier before the reflexive signal was active). This indicates that the learning process was complete, and the reflexive signal no longer occurred. By conducting this learning process at different maximum flying speeds, we obtained an appropriate predictive weight for each maximum speed in relation to its flying speed and the setup of predictive and reflexive ranges. For example, at a faster flying speed, the predictive weight was larger, causing the UAV to brake sharply and stop at a safe distance from the obstacle. This implies that the strength of the predictive weight is a factor or gain in defining the speed adaptation rate of the UAV. Importantly, the neural control and learning system is general and can be applied for speed adaptation in any direction (x, y, or z).

### 2.3. UAV Behavior Control

The UAV behaviors were generated through the output mapping module, which consisted of two sub-modules: speed adaptation and reactive obstacle avoidance output mapping. Speed adaptation output mapping was used to convert the output of N_2_ into the flying speed of the UAV. The UAV flew forward when the output of the module was positive and backward when it was negative. The speed mapping was designed using four control rules, as expressed in Equation (4). The first rule was applied when the output of N_2_ was less than 0.3, and the UAV flew at a maximum speed. The second rule was applied when the output of N_2_ was greater than or equal to 0.3, but less than 0.9, and the UAV reduced its flying speed by the proportional square root of the maximum speed. The third rule was applied when the output of N_2_ was greater than or equal to 0.9, but less than 0.95, and the UAV speed was set to zero. The last rule was applied when the output of N_2_ was greater than or equal to 0.95, and the UAV flew backward at 20% of the maximum speed. The final mapped speed command was applied to the velocity control through a low-level flight controller (Pixhawk/PX4) to control the flying speed of the UAV in the horizontal/vertical plane. Based on this setup, the UAV proactively adapted its flying speed while approaching the obstacle and flew backward if it became too close. This allows other advanced functions (such as obstacle avoidance) to be executed at a safe distance.


(4) $$\begin{array}{l} v_{x,y,z}(t)=\{\begin{array}{ll} v_{x,y,z\_max} & o_{2}(t)<0.3,\\ \sqrt{(1-o_{2}(t))v_{x,y,z\_max}} & 0.3\leq o_{2}(t)<0.9,\\ 0 & 0.9\leq o_{2}(t)<0.95,\\ -0.2v_{x,y,z\_max} & o_{2}(t)\geq 0.95, \end{array} \end{array}$$

where *v*_*x,y,z*_(*t*) denotes the flying speed command for the UAV in the x, y, or z direction; *v*_*x,y,z*_*max*_ is the maximum flying speed of the UAV in the x, y, and z directions; and *o*_2_(*t*) is the output of N_2_. Note that these four rules with the threshold numbers (0.3, 0.9, and 0.95) were empirically defined. While the simple mapping with discrete speed transition is sufficient for the UAV control. To smoothen speed transition, one can apply an advanced technique, such as fuzzy logic (Hartono and Nizar, 2019).

For reactive obstacle avoidance control, because it was not the main focus of this study, we utilized the output of N_2_ as a command to activate obstacle avoidance. As described above, before the UAV performs obstacle avoidance, it should be at a safe distance from the obstacle by reducing its speed and braking, in accordance with the speed adaptation algorithm. The UAV then performs obstacle avoidance, driven by a preprogrammed reactive obstacle avoidance control.

## 3. Experiments and Results

**Figure 4:**
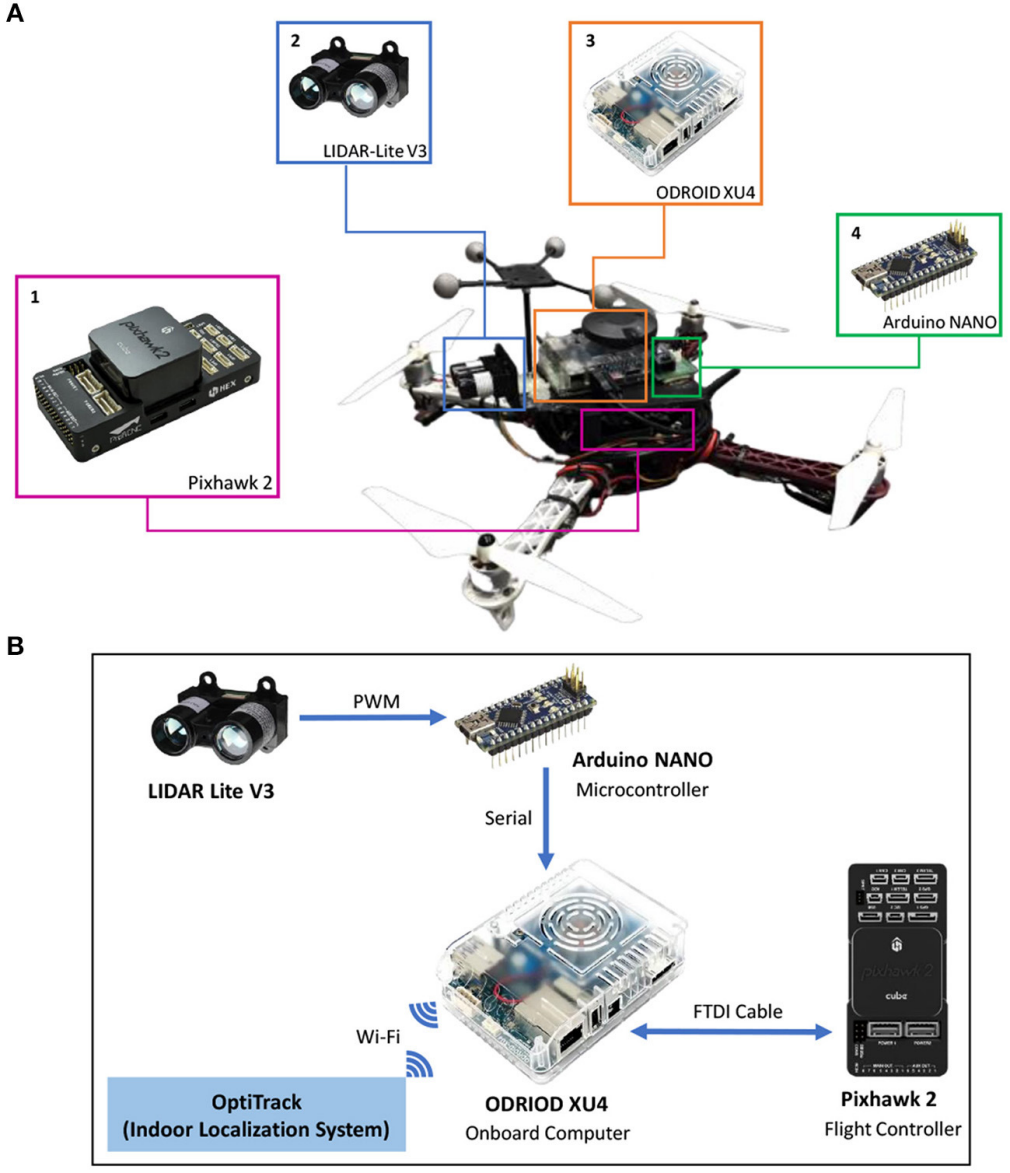
**(A)** The UAV with (1) Pixhawk 2.1 flight controller and its accessories, (2) distance sensor, (3) Odroid onboard computer, and (4) Arduino NANO microcontroller. **(B)** Schematic connection of the setup.

Speed adaptation and obstacle avoidance were implemented on the UAV, as shown in Figure 4A. We used a frame wheel 450 quadrotor (F450) as our experimental UAV platform, where the distance between the diagonal motors of the UAV was 450 mm. The UAV had four brushless, 920 kV motors each attached with a 30 A electronic speed controller (ESC). In Figure 4A, the key components comprise the following: (1) Pixhawk 2.1 (an open-source flight controller) and its accessories, including telemetry for data exchange with a ground station and receiver for obtaining manual control signals from a remote controller; (2) a compact optical distance measurement sensor (LiDAR-Lite V3) for obstacle detection; (3) an onboard computer (Odroid XU4) for implementing the proactive neural controller (Figure 3) and communicating with Pixhawk, Arduino, and the indoor localization system (OptiTrack); and 4) the Arduino NANO microcontroller, serving as buffer hardware for the interface between the sensor and onboard computer. The Odriod onboard computer is the center of all communications, as shown in Figure 4B. The interface between the onboard computer and flight controller was based on the robot operating system (ROS) using a supported bridge between ROS and the MAVLink protocol, called MAVROS, through an FTDI cable. In another ROS interface, located between the onboard computer and localization system, we used the virtual-reality peripheral network (VRPN) library to publish the positions and orientations from the localization system to the UAV using WiFi. The onboard computer was linked to the Arduino through a serial interface using a USB cable, converting the PWM signal from the sensor into a distance and then sending it to the onboard computer.

**Figure 5:**
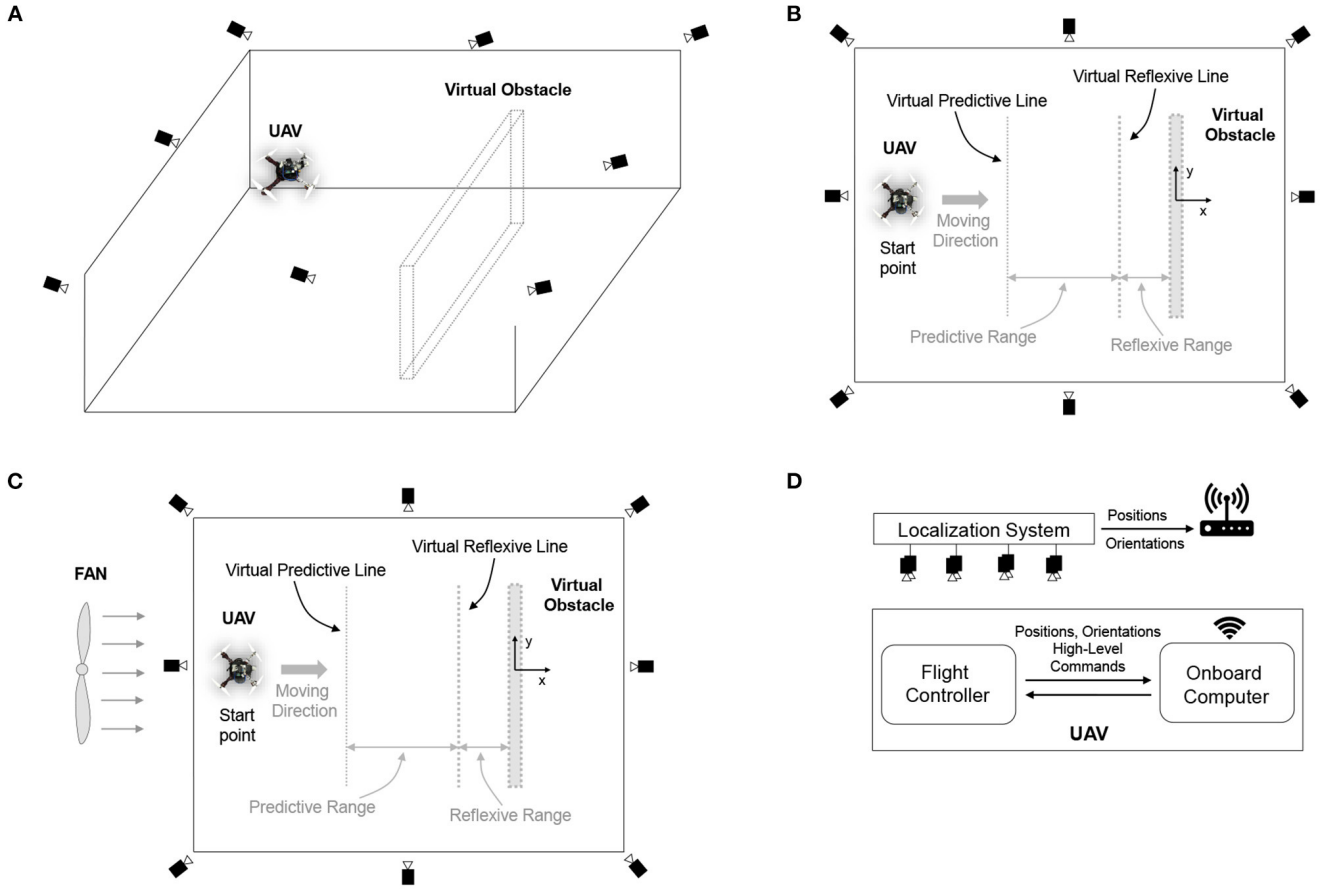
Experimental setup for neural control with online learning of a UAV. **(A)** 3D view of the indoor environment for flying the UAV under the localization system, consisting of eight motion capture cameras. **(B)** Top view of the flying environment without wind perturbation, showing predictive and reflexive ranges with respect to the virtual obstacle. In this setup, the virtual obstacle was placed at position x = 0.0, with virtual predictive and virtual reflexive lines located at x = −3.0 m and −0.5 m, respectively. Basically, the predictive range was 2.5 m, and the reflexive range was 0.5 m. The UAV started flying at position x = −4.5 m. **(C)** Top view of the flying environment with wind perturbation generated by a fan behind the UAV. **(D)** The communication system provided UAV positions (i.e., x, y, z) and orientations (i.e., roll, pitch, yaw) from the localization system to the onboard computer. The communication flow is that the localization system tracks the motion of the UAV and then sends positions and orientations to the onboard computer. The onboard computer transfers positions and orientations to the flight controller for fusing with IMU information. Subsequently, the fused information (positions and orientations) is sent back to the onboard computer to compute and generate control commands.

The following experiments were performed to (1) evaluate the performance of the neural control and online learning for speed adaptation and (2) demonstrate the use of speed adaptation for obstacle avoidance of the UAV. The control system was implemented on the onboard computer at an update frequency of 10 Hz. The first experiment was for the UAV to learn the speed control parameter (predictive weight) online at different flying speeds, which can be separated into two parts (i.e., horizontal and vertical planes). We first learned the weight in the horizontal plane using the experimental setup shown in Figure 5. Because the UAV is symmetric, the horizontal plane experiment will only be conducted in one direction (i.e., the x direction), and the learned weight can be applied for speed adaptation control in both x and y directions. Note that the vertical position of the UAV was maintained at a certain height during the horizontal experiment. To execute the learning process, the UAV flew toward a virtual obstacle at a maximum speed of 0.5 m/s in the real environment setting, as shown in Figure 5B. The distance from the UAV to the virtual obstacle was defined by subtracting the position on the x-axis of the UAV and the virtual obstacle, both of which were provided by the localization system. Instead of using a signal from the distance sensor, this information was used as an input signal for this learning process^5^. The input signal was fed into the neural control to produce the output signal and control the UAV to adapt its flying speed. During the learning process, the algorithm updated the weight based on the online learning mechanism (see Equation 3).

**Figure 6:**
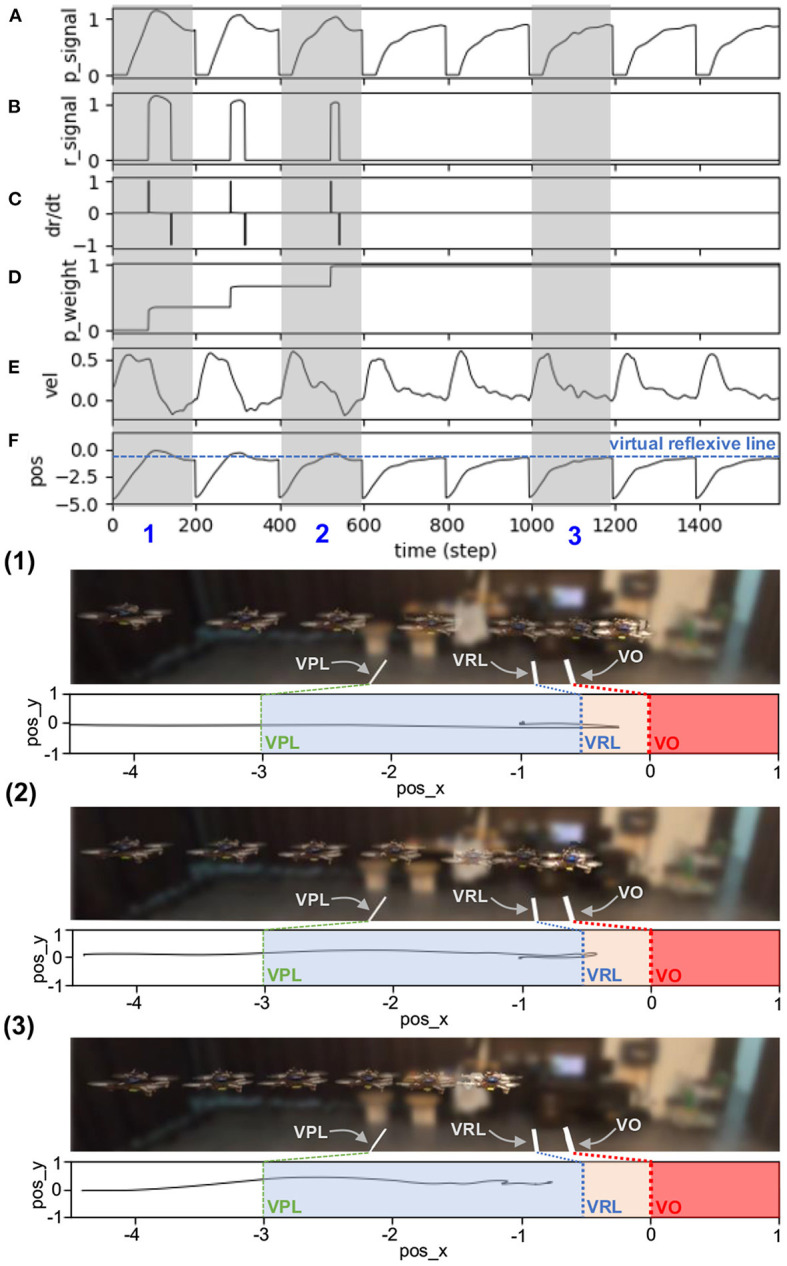
Real-time data of speed adaptation during online learning while flying the UAV at a maximum speed of 0.5 m/s. **(A)** Predictive signal. **(B)** Reflexive signal. **(C)** Derivative of the reflexive signal. **(D)** Predictive weight. **(E)** Actual speed (x-axis) of the UAV. **(F)** Position on the x-axis of the UAV compared to the virtual reflexive line (blue line). The gray areas represent three periods and show the UAV's position with respect to the virtual predictive line (VPL), virtual reflexive line (VRL), and virtual obstacle (VO). These three periods are shown in the three snapshots together with the corresponding estimated trajectories (1, 2, 3) namely the beginning of learning (1), during learning (2), and after learning (3). A video of this experiment can be viewed on www.manoonpong.com/DSA/video1.mp4.

**Figure B1:**
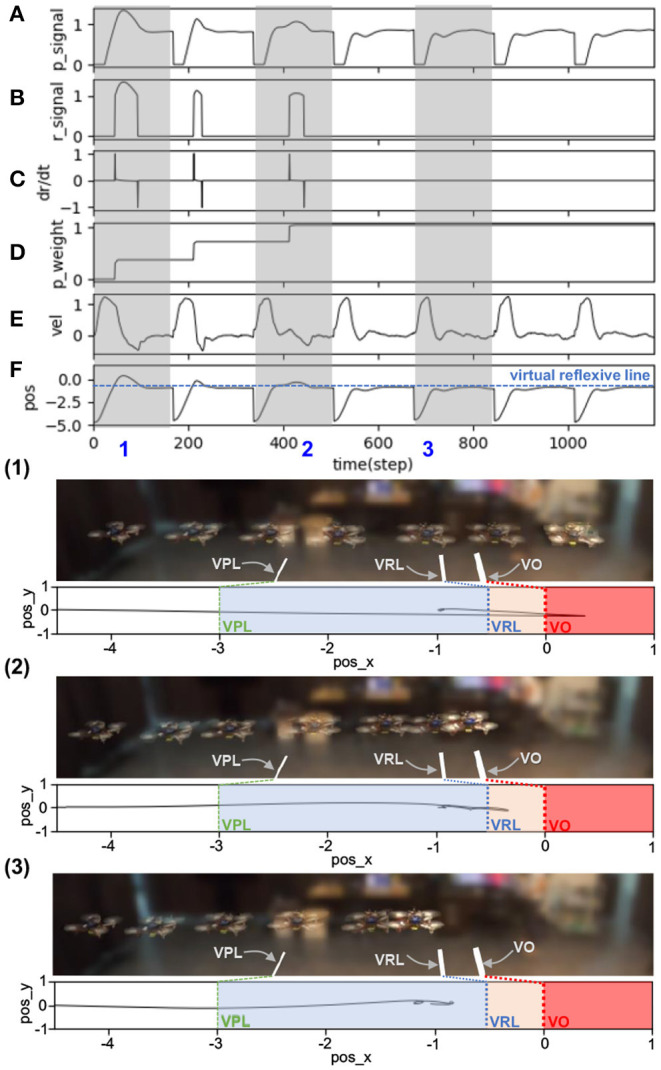
Real-time data of speed adaptation during online learning while flying the UAV at a maximum speed of 1.0 m/s. **(A)** Predictive signal. **(B)** Reflexive signal. **(C)** Derivative of the reflexive signal. **(D)** Predictive weight. **(E)** Actual speed (x-axis) of the UAV. **(F)** Position on the x-axis of the UAV compared to the virtual reflexive line (blue line). The gray areas represent three periods and show the UAV's position with respect to the virtual predictive line (VPL), virtual reflexive line (VRL), and virtual obstacle (VO). These three periods are shown in the three snapshots together with the corresponding estimated trajectories (1, 2, 3) namely the beginning of learning (1), during learning (2), and after learning (3).

**Figure B2:**
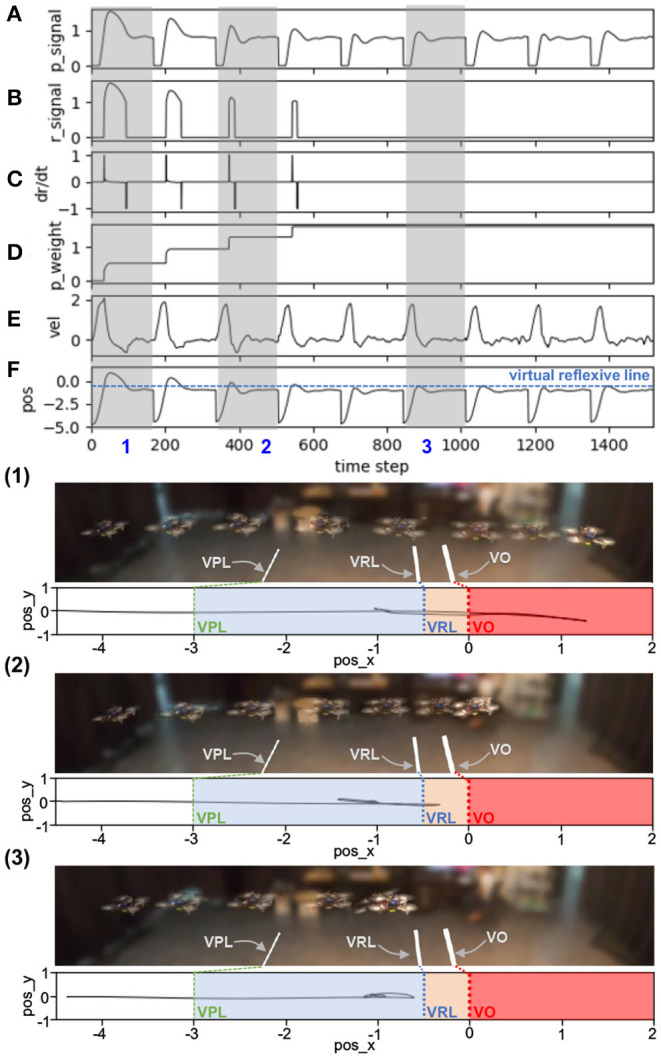
Real-time data of speed adaptation during online learning while flying the UAV at a maximum speed of 1.5 m/s. **(A)** Predictive signal. **(B)** Reflexive signal. **(C)** Derivative of the reflexive signal. **(D)** Predictive weight. **(E)** Actual speed (x-axis) of the UAV. **(F)** Position on the x-axis of the UAV compared to the virtual reflexive line (blue line). The gray areas represent three periods and show the UAV's position with respect to the virtual predictive line (VPL), virtual reflexive line (VRL), and virtual obstacle (VO). These three periods are shown in the three snapshots together with the corresponding estimated trajectories (1, 2, 3) namely the beginning of learning (1), during learning (2), and after learning (3).

The results in Figure 6 show real-time data during online learning at a maximum speed of 0.5 m/s. Initially, the UAV flew forward toward the obstacle, causing a decrease in the distance between the two. Once the UAV flew into the predictive range, the predictive signal became active (Figure 6A). The UAV still flew toward the obstacle until it reached the reflexive range. Once the UAV was in the reflexive range, the reflexive signal became active (Figure 6B). The predictive weight gradually increased (Figure 6D) owing to the correlation of the predictive signal and a positive change in the reflexive signal (i.e., a positive value of the derivative of the reflexive signal), as described in Equation (3). Consequently, the N_2_ output was activated based on a combination of predictive and reflexive signals. This caused the speed command in the x-axis to decrease, as described in Equation (4) (Figure 6E). However, because the predictive weight was initialized with zero, the UAV speed in this state decreased only because of the reflexive signal. During the first trial, the UAV speed started to decrease speed and braked after passing through the virtual reflexive line. Although the system performed the same process in the following trials, the UAV started to proactively adapt its speed. This is because the predictive signal contributes to the speed adaptation through the learned predictive weight. In each trial, the system started with the previous (learned) predictive weight; for example, in the first trial, the predictive weight was initially set to 0.0, whereas in the second trial, it started with the learned value from the previous trial (Figure 6D). During the second trial, the UAV reacted earlier (proacted) by adapting its speed before passing the virtual reflexive line, owing to the influence of the increased predictive weight. The process was automatically and continuously executed such that the UAV gradually proacted before passing through the line. After three learning trials, the UAV successfully adapted its flying speed proactively and braked before hitting the line. At this point, the reflexive signal did not occur. Consequently, the predictive weight did not increase further or converged. The UAV behaved in the same manner in the remaining trials after learning (see the last five trials (from 600 time steps) in Figures 6A,F). This implies that the learning process was completed, with the existing predictive weight being the optimal control parameter for a flying speed of 0.5 m/s. Moreover, we showed the UAV behavior in three periods as snapshots during the learning process, as represented by the gray areas in Figure 6(1)–(3). The same learning process was performed for the other maximum flying speeds (0.7, 0.9, 1.0, 1.1, 1.3, and 1.5 m/s). The results at speeds of 1.0 and 1.5 m/s are presented in Appendix B (Figures B1,B2).

**Figure 7:**
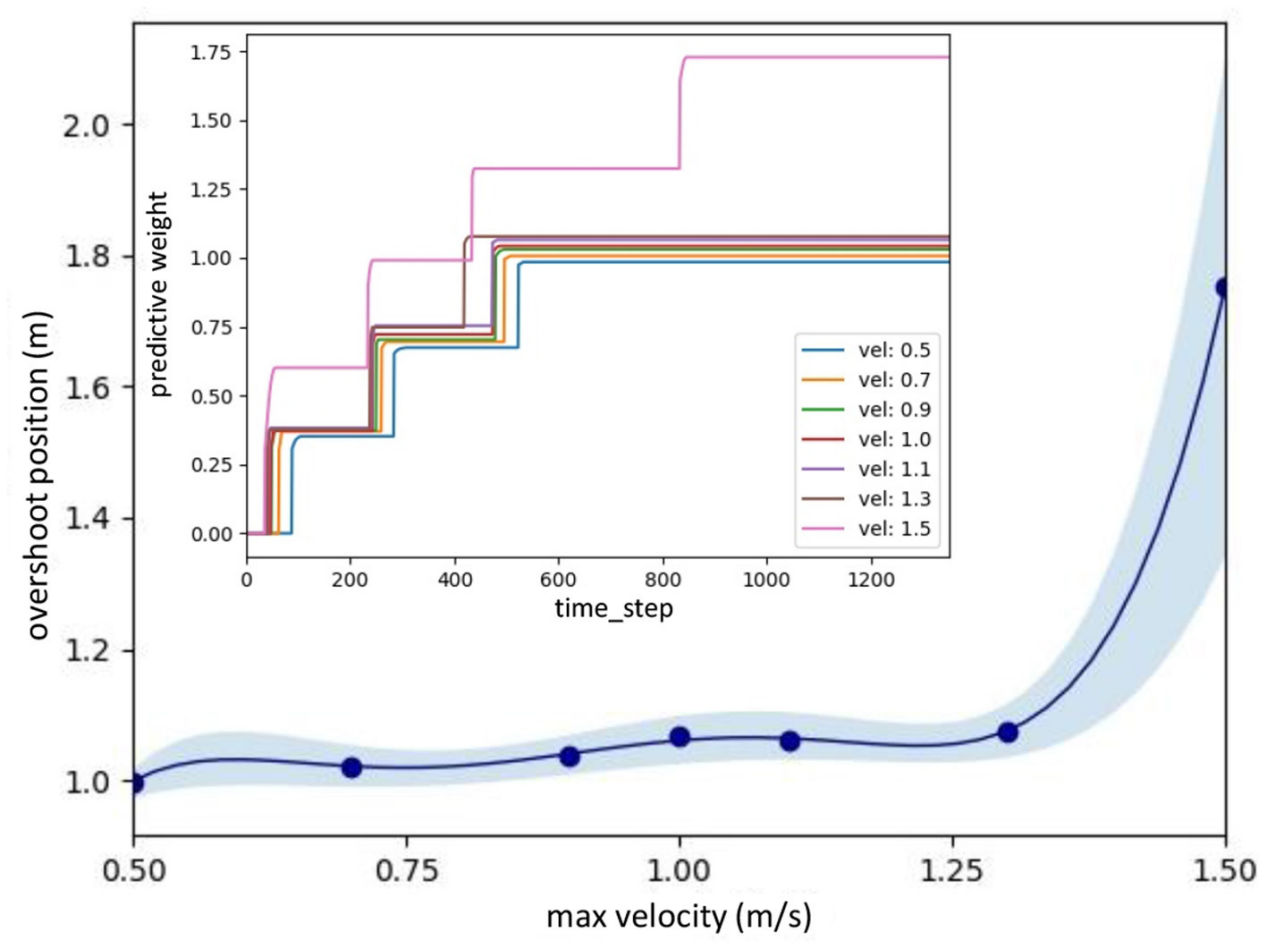
Approximated predictive weights based on the seven learned weights at the maximum flying speeds of 0.5, 0.7, 0.9, 1.0, 1.1, 1.3, and 1.5 m/s. For the seven speeds, we repeated the learning process seven times each. The inset illustrates the learning curves of predictive weights at the maximum flying speeds. It can be seen that the learning process was fast where the weights converged after 3–4 trials.

**Figure 8:**
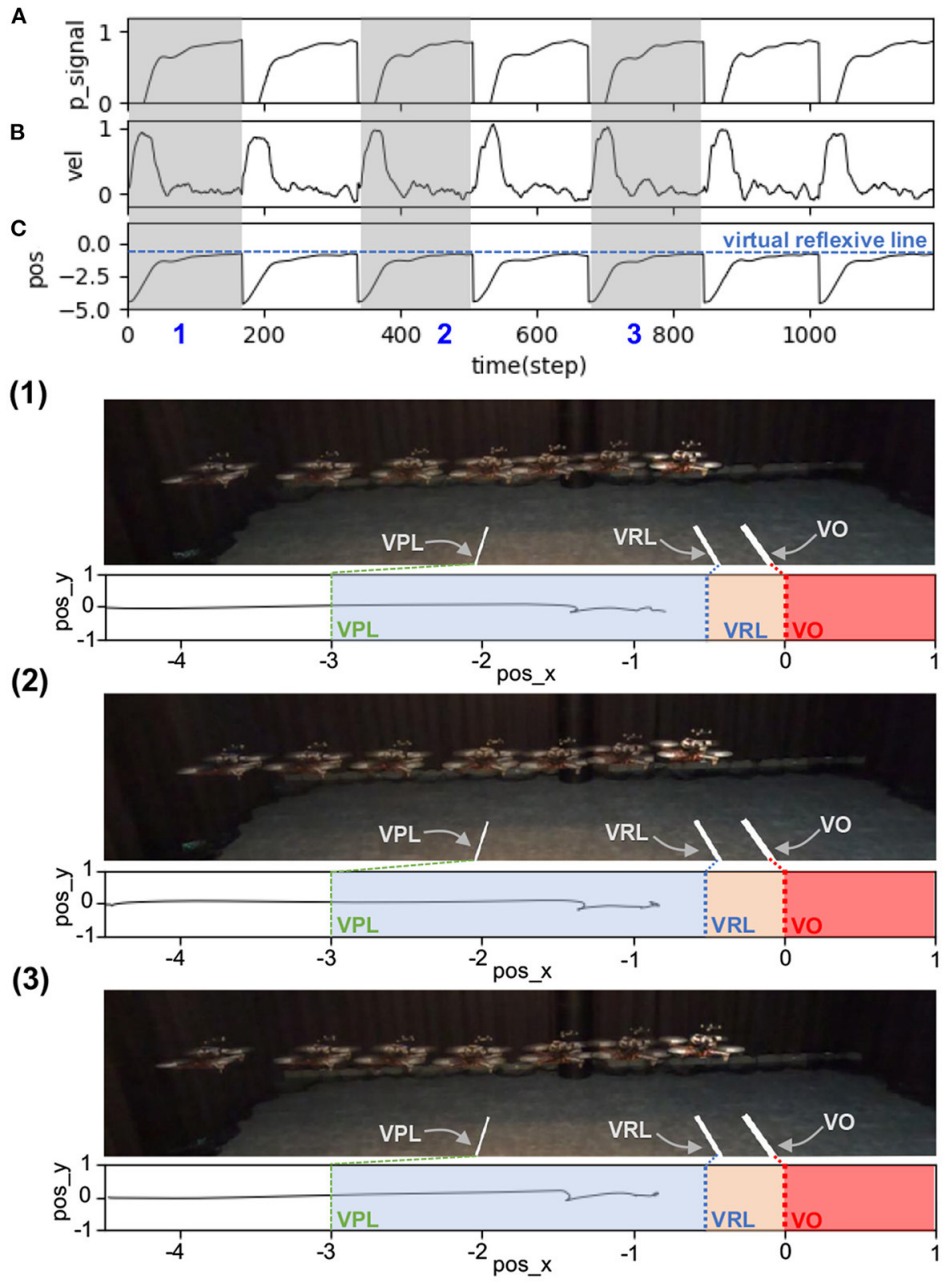
Real-time data when flying the UAV at a maximum speed of 0.75 m/s and using an approximated predictive weight of 1.03 (obtained from Figure 7) as the initial weight. Each graph shows: **(A)** predictive signal, **(B)** actual speed (x-axis) of the UAV, and **(C)** position on the x-axis of the UAV compared to the virtual reflexive line (blue line). The gray areas represent three periods, showing the position of the UAV with respect to the virtual predictive line (VPL), virtual reflexive line (VRL), and virtual obstacle (VO) in three snapshots together with the corresponding estimated trajectories (1, 2, 3).

**Figure 9:**
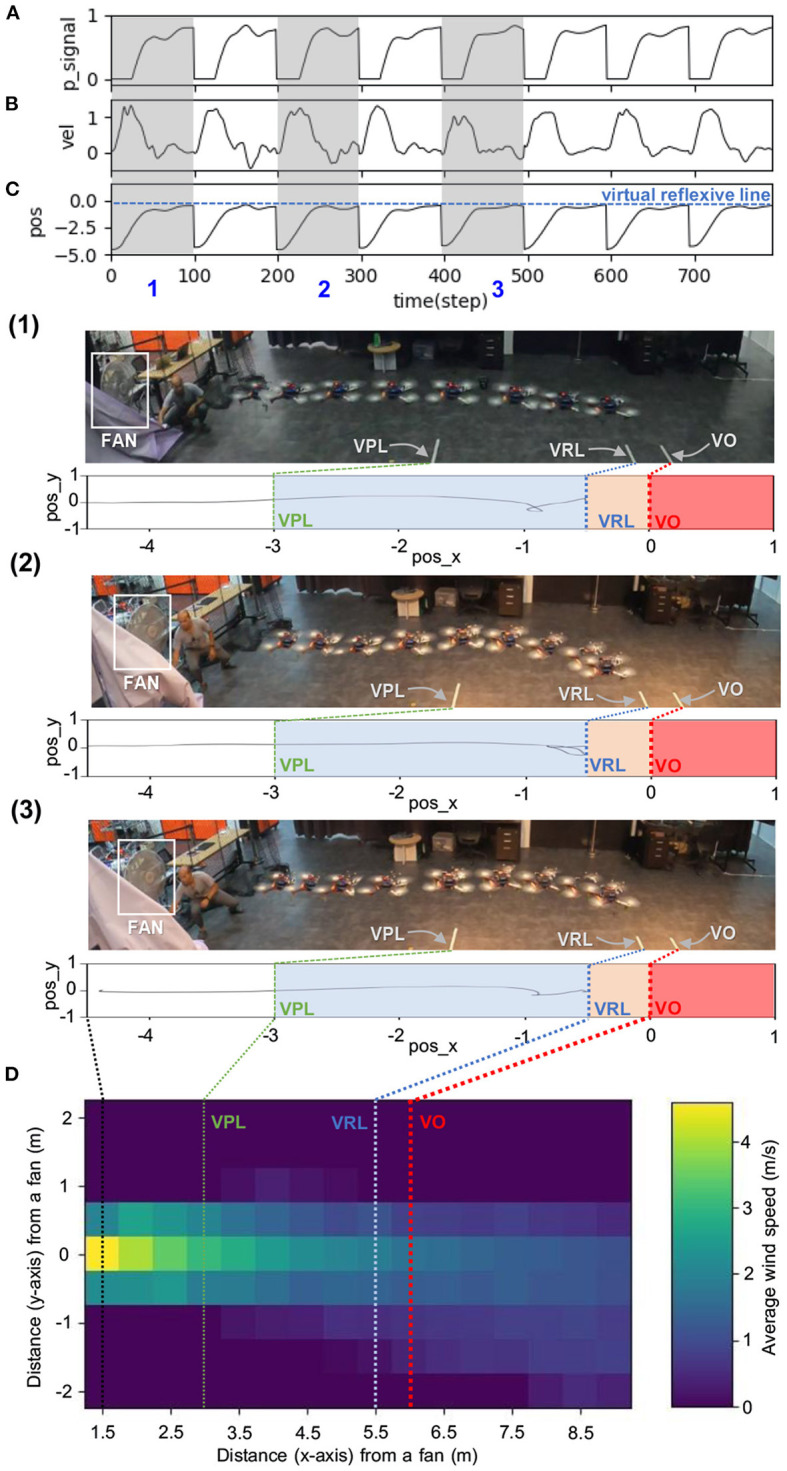
Real-time data when flying the UAV with wind perturbation. The UAV flew at a maximum speed of 1.0 m/s and used an approximated predictive weight of 1.07 (obtained from Figure 7) as the initial weight. Each graph shows: **(A)** predictive signal, **(B)** actual speed (x-axis) of the UAV, **(C)** position on the x-axis of the UAV compared to the virtual reflexive line (blue line), and **(D)** wind speed gradient. The gray areas represent three periods, showing the position of the UAV with respect to the virtual predictive line (VPL), virtual reflexive line (VRL), and virtual obstacle (VO) in three snapshots together with the corresponding estimated trajectories (1, 2, 3). A video of this experiment can be viewed on www.manoonpong.com/DSA/video2.mp4.

**Figure 10:**
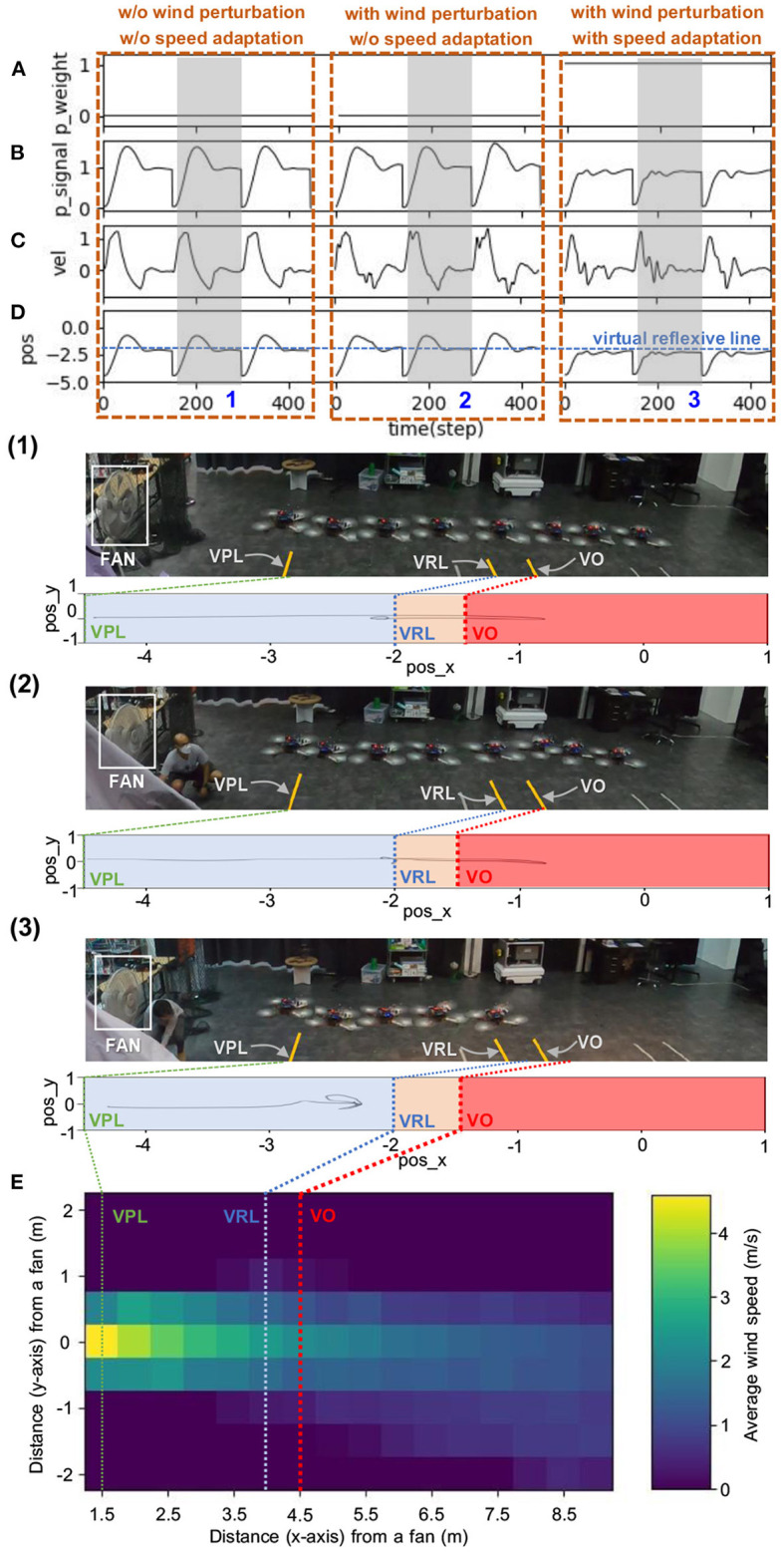
Real-time data when flying the UAV with strong wind perturbation. The UAV flew at a maximum speed of 1.0 m/s. Each graph shows: **(A)** predictive weight, **(B)** predictive signal, **(C)** actual speed (x-axis) of the UAV, **(D)** position on the x-axis of the UAV compared to the virtual reflexive line (blue line), and **(E)** wind speed gradient. The three brown dashed blocks are the three different flying conditions: (1) without (w/o) wind perturbation and without (w/o) speed adaptation, (2) with wind perturbation and without (w/o) speed adaptation, and (3) with wind perturbation and with speed adaptation). The gray areas represent three periods, showing the position of the UAV with respect to the virtual predictive line (VPL), virtual reflexive line (VRL), and virtual obstacle (VO) in three snapshots, together with the corresponding estimated trajectories (1, 2, 3). A video of this experiment can be viewed on www.manoonpong.com/DSA/video3.mp4.

**Figure C1:**
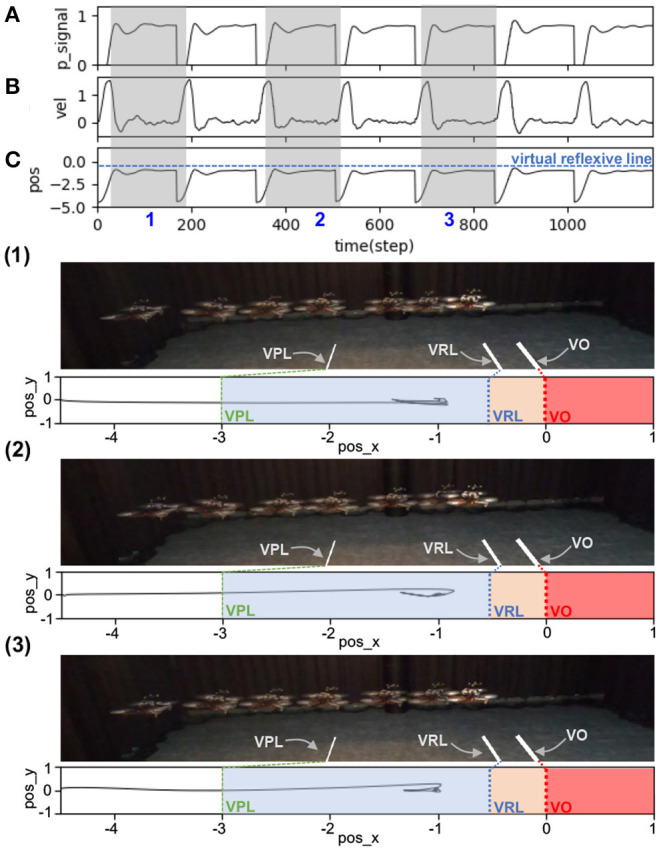
Real-time data when flying the UAV at a maximum speed of 1.25 m/s and using an approximate predictive weight of 1.07 obtained from Figure 7 as the initial weight. Each graph shows: **(A)** predictive signal, **(B)** actual speed (x-axis) of the UAV, and **(C)** position on the x-axis of the UAV compared to the virtual reflexive line (blue line). The gray areas represent three learning periods and show the UAV's position with respect to the virtual predictive line (VPL), virtual reflexive line (VRL), and virtual obstacle (VO), as in three snapshots together with the corresponding estimated trajectories (1, 2, 3).

The statistical data for the speed adaptation algorithm are shown in Figure 7. It was calculated using polynomial regression to fit the relationship between the optimal predictive weights and the UAV's corresponding maximum flying speeds (0.5, 0.7, 0.9, 1.0, 1.1, 1.3, and 1.5 m/s). In this case, the experiment was repeated seven times at each speed to observe the distribution of the optimal predictive weight. The graph shows the average, minimum, and maximum values of each predictive weight. Moreover, the results show the approximated predictive weights for other flying speeds between 0.5 and 1.5 m/s. To verify the usability of the approximated weights, we chose two other maximum flying speeds: 0.75 and 1.25 m/s. We then performed the same experiment by flying the UAV toward the virtual obstacle using the approximated predictive weights of 1.03, and 1.07, respectively, obtained from Figure 7 at the speeds of 0.75 and 1.25 m/s as the initial weights. Verification results at a maximum speed of 0.75 m/s are shown in Figure 8. The UAV adapted its speed and braked before hitting the virtual reflexive line. Furthermore, the predictive weight did not increase during the experiment, indicating that the weight used was appropriate for this flying speed. Similar results for the 1.25 m/s speed are shown in Appendix C (Figure C1). Both experiments proved that the approximated predictive weights are usable for the speed adaptation function of the UAV when flying at different maximum speeds. Additionally, to demonstrate the robustness of our neural proactive control, we tested it with wind perturbation (see the experimental setup in Figure 5C). In this experiment, we flew the UAV at a maximum speed of 1.0 m/s and used the learned predictive weight of 1.07, obtained from Figure 7. To generate wind perturbation, we placed a fan 1.5 m behind the UAV. The wind gradient is shown in Figure 9D. Figure 9 shows that our control can successfully adapt the flying speed of the UAV and brake it at a safe distance from the virtual obstacle. To investigate the greater effect of wind perturbation on the UAV, we shifted the UAV and all virtual lines (predictive, reflexive, and obstacle) backward by 1.5 m (Figure 10E). Consequently, the UAV has to brake earlier while receiving strong wind perturbation. Figure 10 shows a comparison of the UAV flying behavior under three different conditions. The first condition was when flying the UAV without wind perturbation and without speed adaptation^6^. The second condition was when flying the UAV with wind perturbation and without speed adaptation. The third was when flying the UAV with wind perturbation and with speed adaptation (using an initial predictive weight of 1.07 (obtained from Figure 7) and allowed it to online learn). It can be seen that the wind perturbation strongly affected the UAV velocity control where without speed adaptation, the UAV could not brake and flew beyond the virtual obstacle (Figures 10C,D). In contrast, using our speed adaptation method, the UAV managed to effectively brake without hitting the virtual obstacle.

**Figure 11:**
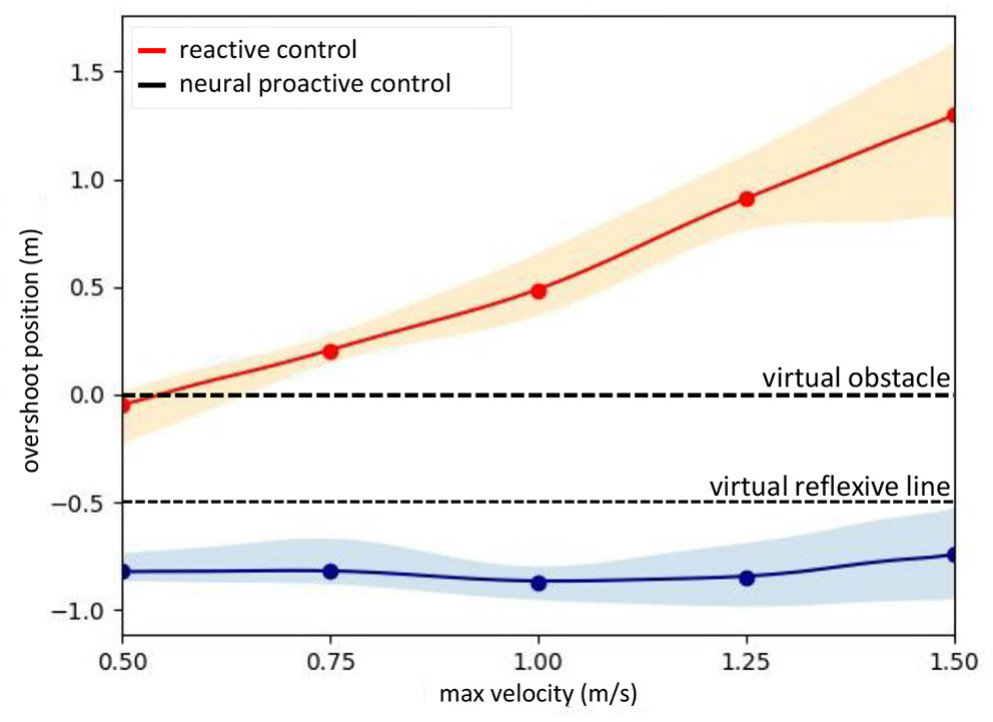
Performance comparison of the real UAV (Figure 4) when flying with neural proactive control (blue) and conventional reactive control (orange). The graph was plotted using the maximum overshoot position of the UAV while flying at different maximum speeds. The proactive control used the predictive signal with the optimal learned predictive weights or gains (obtained from Figure 7) while the reactive control used the reflexive signal with a fixed weight or gain (i.e., 1.0) for all speeds. To observe the distribution of the system we repeated seven times for each speed.

A further performance comparison between the UAV flying with and without the speed adaptation at different flying speeds is presented in Figure 11. The graph was plotted from the maximum flying speeds of the UAV and the maximum overshoot position reached by the UAV before braking and returning to the desired point (the front of the reflexive line). The data were recorded during the experiment by flying at five different speeds (i.e., 0.5, 0.75, 1.0, 1.25, and 1.5 m/s) and repeated seven times for each speed. We used polynomial regression to approximate the data at other flying speeds. The results show that the UAV flying with the proactive control can adapt its flying speed and stop before hitting the virtual reflexive line. While flying with the reactive control or using the conventional reactive control, the UAV was unable to stop before hitting the virtual reflexive line, and at a speed higher than approximately 0.6 m/s, it hit or flew beyond the virtual obstacle line. This implies that the UAV flying in the horizontal plane with reactive control or inappropriate gain will collide with the obstacle when flying in a real environment.

**Figure 12:**
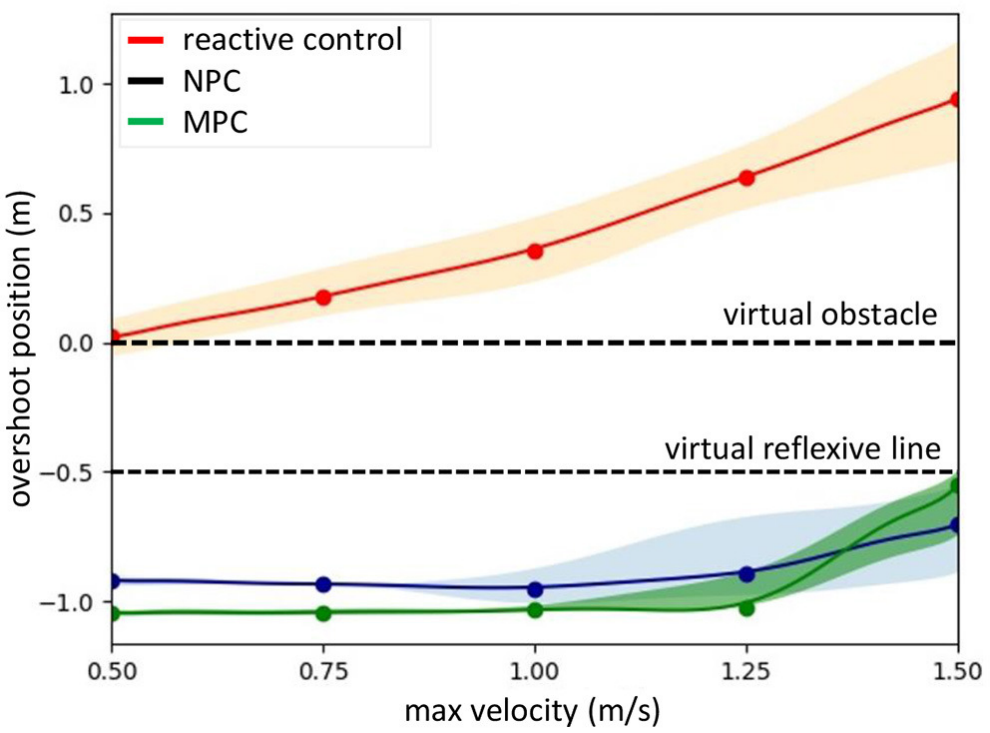
Performance comparison of a simulated IRIS UAV (Supplementary Figure 1) when flying with speed control using the neural proactive control (NPC, blue), MPC (green), or conventional reactive control (orange). The graph was plotted using the maximum overshoot position of the UAV while flying at different maximum speeds. To observe the distribution of the system, we repeated seven times for each speed.

We further performed the same experiment in a simulated environment (Gazebo) to show the ability to apply to another UAV system without requiring its dynamic model or other specific parameter setup (see Supplementary Material). In addition, we compared our model-free neural proactive control technique with the model-based explicit MPC technique (Varshney et al., 2019; Lindqvist et al., 2020; Wang et al., 2021) in the same simulated environment. The simulation results are shown in Figure 12. Clearly, when flying with the conventional reactive control, the UAV was unable to stop before hitting the virtual reflexive line. In contrast, when flying with neural control or MPC, the UAV was able to adapt its speed to brake before hitting the reflexive line. Nevertheless, as the maximum flying speed increased (i.e., 1.5 m/s), the UAV with the MPC had difficulty adapting its speed. Consequently, its braking position was significantly close to the reflexive line; while using neural control, the UAV still adapted its speed and braked at a certain distance before the line (Figure 12). Comparing our neural control with the MPC, there are more system requirements and parameter setups for implementing the MPC, such as the dynamic model of the UAV and system states (e.g., distance from obstacle or position, speed, and acceleration). Moreover, the optimization process of the MPC requires high computational effort. In contrast, the proposed neural control method requires only a distance from an obstacle or position with a few learning trials (less computational effort). The MPC implementation is provided in Supplementary Material. We also provide a detailed comparison of the MPC and neural proactive control (NPC) in Table 1.

In addition to the horizontal plane experiments described above, we further investigated the speed adaptation when flying upward and downward in the vertical plane (Figure 2, left). In this case, the learned predictive weights of the horizontal plane (Figure 7) cannot be used directly for speed adaptation in the vertical plane. Furthermore, a predictive weight for flying upward cannot be used effectively for flying downward and vice versa. This is because of the asymmetrical structure of the UAV along the vertical axis and the force of gravity that resists and pulls down the UAV during upward and downward flying, respectively. Thus, we let real and simulated UAVs (Figure 4 and Supplementary Figure 1) learn the predictive weights for different maximum speeds in the upward and downward flying directions. The experimental setup and results are presented in Supplementary Material. A video of the real UAV experiment is available at www.manoonpong.com/DSA/video4.mp4

**Figure 13:**
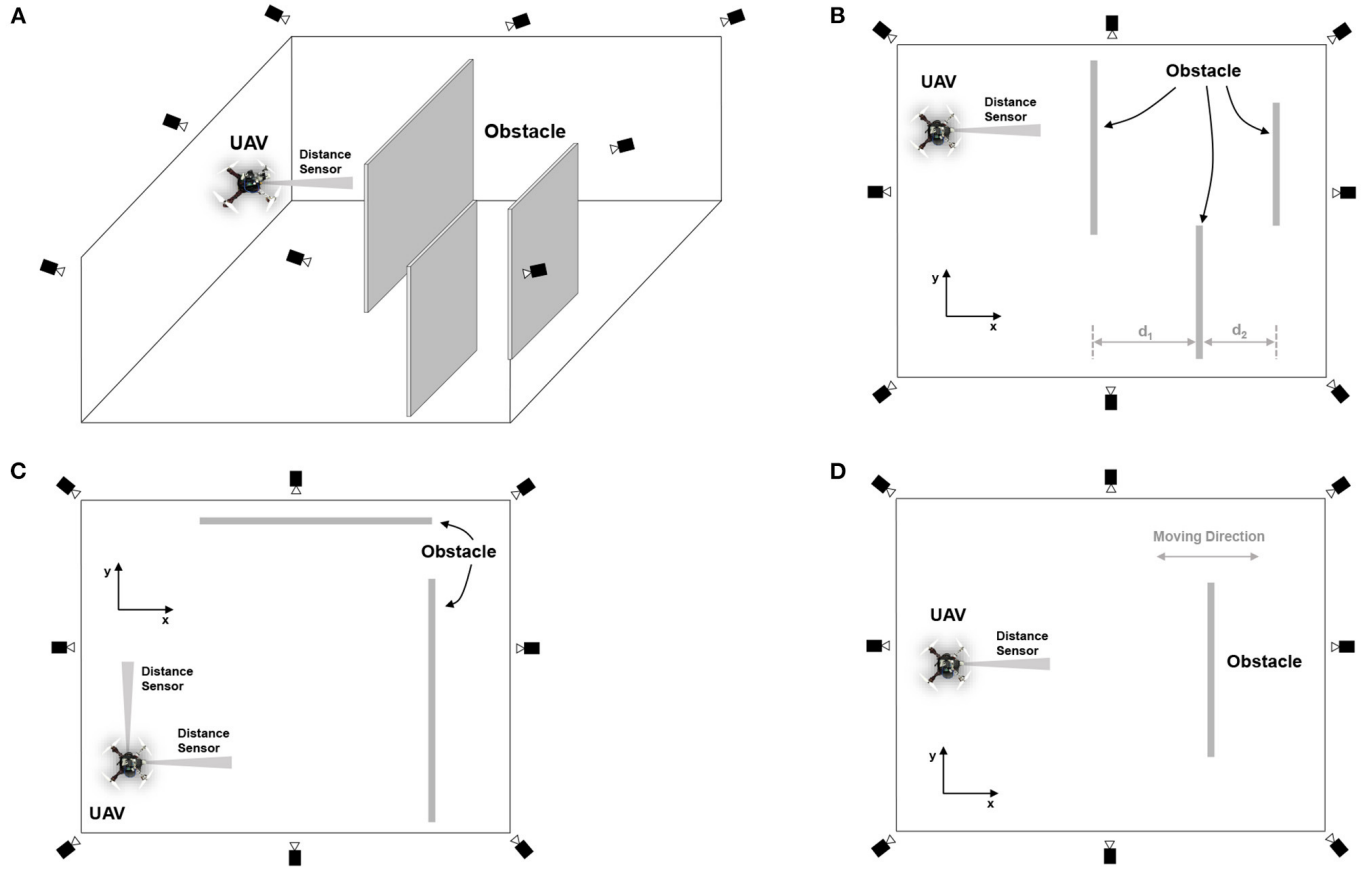
Experimental setup for speed adaptation and reactive obstacle avoidance in the real environment. **(A)** 3D view of the indoor environment for flying the UAV under a localization system, consisting of eight motion capture cameras. **(B)** Top view of the environment, showing the detection ranges and parameter setting when using the speed adaptation algorithm for obstacle avoidance. In this setup, three obstacles were placed in sequence with the distances d1 and d2 set to 2.0 and 1.5 m, respectively. To detect the obstacle, we used the distance sensor installed at the front of the UAV. Note that we used the information obtained from the localization system (motion capture) for the altitude or z position control of the UAV. **(C)** Top view of the environment showing obstacles placed in both x and y directions of the UAV. **(D)** Top view of the environment showing the movable obstacle placed in the x direction of the UAV.

**Figure 14:**
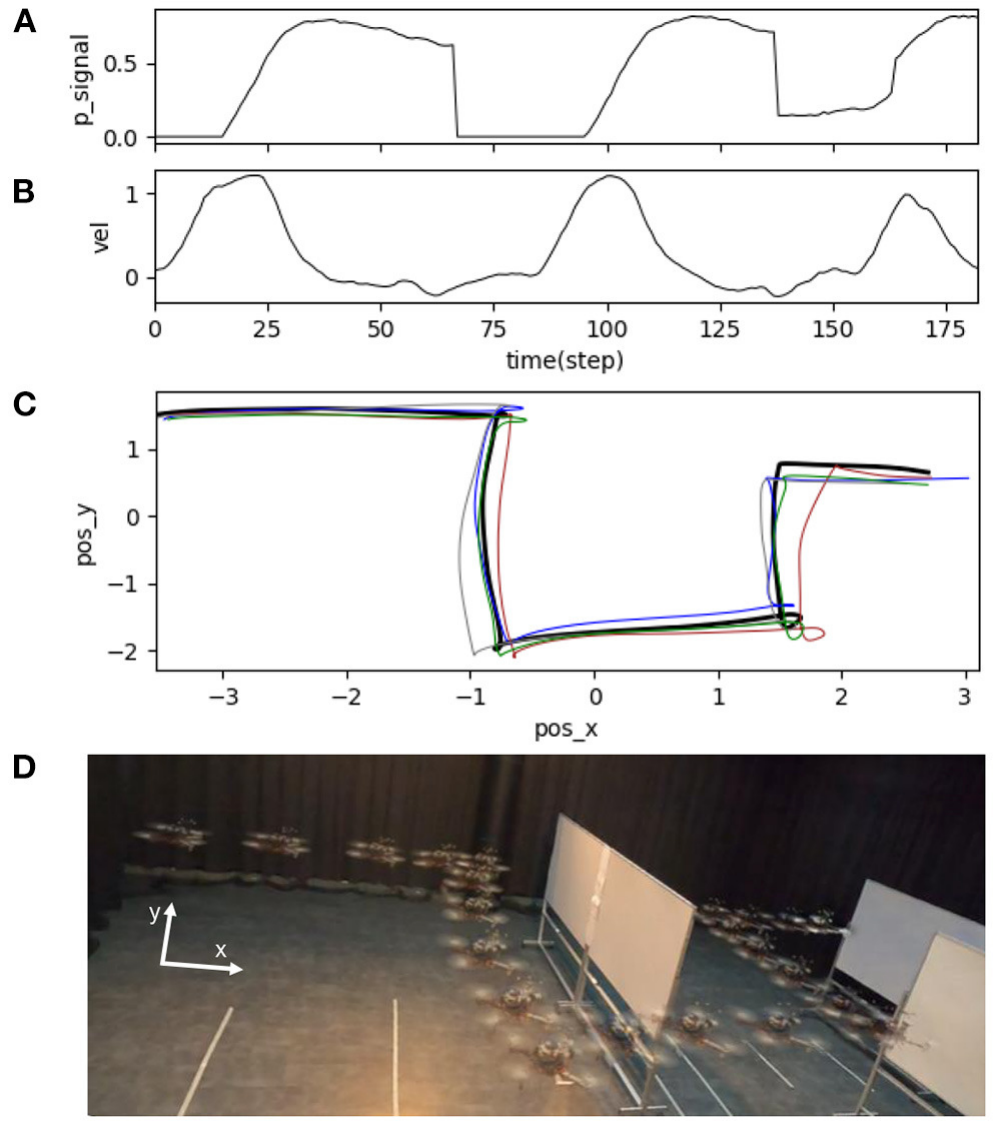
Real-time data of speed adaptation with obstacle avoidance when flying the UAV in the real environment at a maximum speed of 1.0 m/s and a predictive weight of 1.07. **(A)** Predictive signal. **(B)** Actual speed (x-axis) of the UAV. **(C)** Position of the UAV plotting on the x-y axis to show its overall trajectory five times repeatedly. **(D)** Automatic flying behavior of the UAV (see from the left to right). A video of this experiment can be viewed on www.manoonpong.com/DSA/video5.mp4.

In the second experiment, the neural proactive control was used to support reactive obstacle avoidance. We divided the experiment into three sub-experiments. The first sub-experimental setup is shown in Figures 13A,B. In this experiment, a real obstacle detection signal from the distance sensor was used as the input signal of the neural control system. For this test, we programmed the UAV to avoid an obstacle by flying right/left once when the final neural output (N_2_) from the neural proactive control was higher than a threshold (e.g., 0.75). This can be considered as a reactive command to activate obstacle avoidance behavior. The results in Figure 14 show the UAV behavior when flying in the environment with real obstacles at a maximum speed of 1.0 m/s, using the predictive weight of 1.07 (Figure 7). The following behaviors were then performed: (1) the sensor detected the obstacle, (2) the UAV started to adapt its flying speed and proactively braked at a safe distance from the obstacle, (3) the UAV automatically avoided the obstacle with respect to the preprogrammed obstacle avoidance behavior, and (4) the UAV subsequently continued flying forward. Similar behavior was observed when detecting the following obstacle.

**Figure 15:**
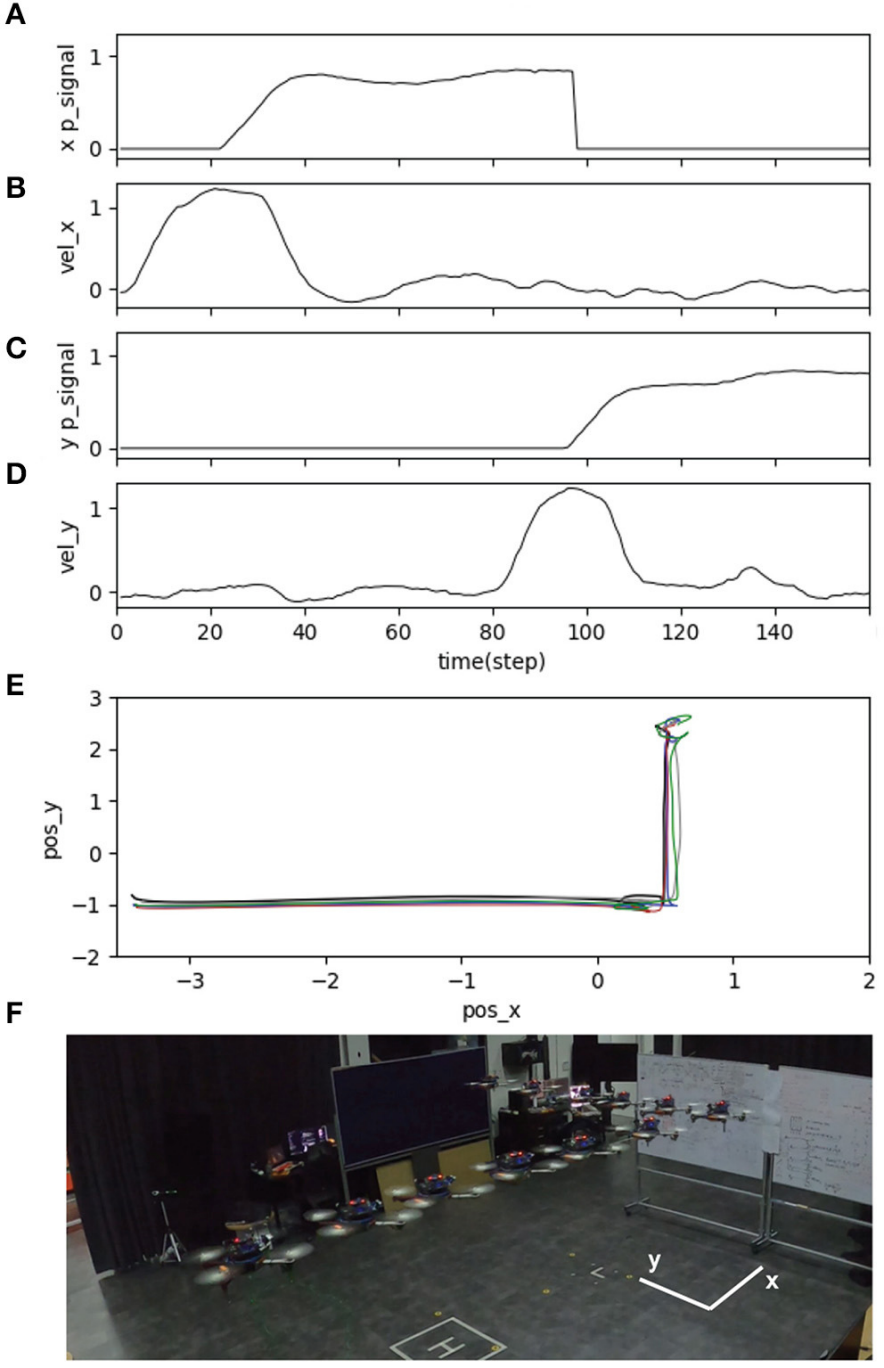
Real-time data of speed adaptation in the forward and sideward directions of the UAV, when flying the UAV in the real environment at a maximum speed of 1.0 m/s and a predictive weight of 1.07. **(A)** Predictive signal from the distance sensor for detecting an obstacle in the forward direction. **(B)** Actual speed in the forward direction. **(C)** Predictive signal from the distance sensor for detecting an obstacle in the sideward direction. **(D)** Actual speed in the sideward direction. **(E)** Position of the UAV plotted on the x-y axis to show its overall trajectory five times repeatedly. **(F)** Automatic flying behavior of the UAV (see from the left to right). A video of this experiment can be viewed on www.manoonpong.com/DSA/video6.mp4.

The second sub-experiment demonstrates the usability of the proposed neural control system for adapting the speed of the UAV in multiple directions (i.e., adapting the speed in the forward (x-axis) and sideward (y-axis) directions). The experimental setup for the test is illustrated in Figure 13C. We regulated the pitch and roll commands of the UAV to adapt the speed in the forward and sideward directions, respectively. In this scenario, we added another distance sensor to detect an obstacle from the side and another neural control and online learning module for sideward-speed adaptation control. We flew the UAV at a maximum speed of 1.0 m/s and used a predictive weight of 1.07 (learned from flying in the forward direction; Figure 7) for both directions. The results in Figure 15 show the UAV's behavior. The UAV first flew forward, adapted its flying speed, and braked proactively at a safe distance from the front obstacle. It then flew sideways to the left and proactively braked at a safe distance from the side obstacle.

**Figure 16:**
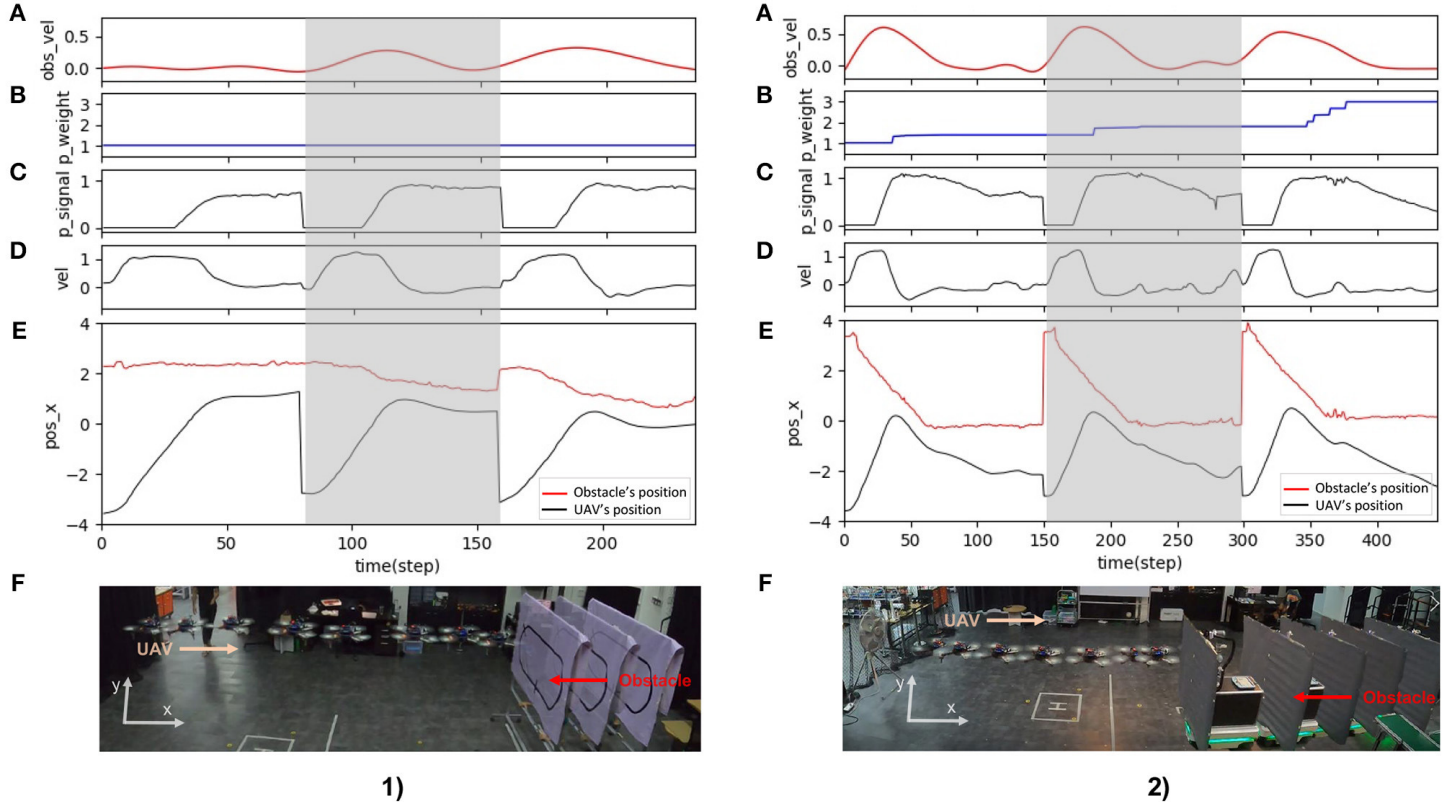
Real-time data of speed adaptation with a dynamic obstacle when flying the UAV in the real environment at a maximum speed of 1.0 m/s and a predictive weight of 1.07. (1) The obstacle speed was approximately 0.25 m/s. The obstacle was moved by a human. (2) The obstacle speed was approximately 0.5 m/s. The obstacle was moved by a mobile robot. **(A)** Actual obstacle speed. **(B)** Predictive Weight. **(C)** Predictive signal. **(D)** Actual speed (x-axis) of the UAV. **(E)** Position on the x-axis of the UAV and obstacle. **(F)** Automatic flying behavior of the UAV (see from left to right) from the gray area period of the above graphs. A video of this experiment can be viewed on www.manoonpong.com/DSA/video7.mp4.

Finally, the third sub-experiment demonstrates the adaptability of the proposed neural control system to tackle a dynamic obstacle. The experimental setup for the test is shown in Figure 13D. We performed this experiment using the same speed and predictive weight as in the second sub-experiment. Here, while the UAV was flying forward, we moved the obstacle at speeds of 0.25 and 0.5 m/s toward it. Figure 16 shows the behavior of the UAV when facing a dynamic obstacle. The UAV successfully adapted its speed without colliding with the obstacle. For the obstacle speed of 0.25 m/s (Figure 16.1), the system managed to adapt the speed of the UAV without any further learning (Figure 16.1B), while the control system automatically performed further learning to adapt the speed of the UAV to deal with the obstacle speed of 0.5 m/s (Figure 16.2). This is because the relative speed of the UAV and obstacle was significantly increased, and the existing predictive weight could not effectively brake the UAV. Consequently, the reflexive signal was triggered, resulting in further learning or predictive weight adaptation (Figure 16.2B). All three sub-experiments (Figures 14–16) were repeated five times to assess the robustness and performance of the system. The results show that the UAV was capable of safely avoiding obstacles on all five occasions. The demonstrations show that the neural proactive controller can be used to control the speed of the UAV in multiple directions as well as deal with a dynamic obstacle. This proves that the speed adaptation algorithm of the neural controller can be used to generate primitive adaptive behavior, or a basis function before the UAV performs more complex behaviors, such as avoiding obstacles (as shown here), approaching certain targets, and following an object at different speeds.

## 4. Discussion

The experiments and results above show how our proposed method works in the training session, prove our system's robustness and generalization on different UAV platforms, and demonstrate the application of our system as the basis function for obstacle avoidance control. Here, we further discuss our proposed method, which in principle consists of a non-neural component and neural system. While our neural system is used for speed control and learning (Figure 3B), the non-neural component is also required to store the learned predictive weights, approximate the weights using polynomial regression (Figure 7), and select a proper predictive weight value with respect to a given maximum flying speed. During online learning, the neural system updates the weight, which is then stored in the non-neural component. Multiple learning sessions were conducted to obtain multiple learned predictive weights with respect to the corresponding maximum flying speeds. After online learning, the polynomial regression process was performed offline to build a relationship between the predictive weights and maximum flying speeds, and then a proper approximated predictive weight was selected with respect to the current maximum speed. Because the control system can continuously learn online, the weight can be adapted to deal with environmental changes. After the new weight value is obtained, it is stored in the non-neural component, and the entire process is repeated. It is important to note that, in this setup, the neural system with online learning is model-free, whereas a part of the non-neural component that uses polynomial regression is model-based.

Furthermore, we briefly discuss some of the remaining issues concerning the neural-based speed adaptation control system of UAVs. The proposed speed adaptation control combines two sensory signals (reflexive and predictive signals), in which each signal can generate different types of behavior. The reflexive signal (short range) with a fixed weight (i.e., 1.0) can produce reactive behavior once the signal is activated. The predictive signal (long range) with an adaptive weight can create adaptive behavior, allowing the UAV to proactively adapt its flying speed once the signal is activated. We correlated these two sensory signals using neural correlation-based learning, which is based on the ICO learning principle. The ICO learning method is typically used in low-dynamical systems or slow-speed systems, such as walking and wheeled robots. It has been used to generate various adaptive behaviors in robots, such as goal-directed navigation (Shaikh and Manoonpong, 2017), up-slope locomotion (Manoonpong and Wörgötter, 2009), acoustic predator-recognition and escape response (Manoonpong and Wörgötter, 2009), and food retrieval tasks (Porr and Wörgötter, 2007). Owing to its stable and fast convergence characteristics and computational efficiency (as shown in Porr and Wörgötter, 2006), ICO learning was exploited here for the first time to control a highly dynamical system, such as a UAV. Typically, for a UAV to fly safely without colliding with an obstacle under a conventional reactive control system, the safe braking distance have to be manually tuned for each maximum flying speed. This problem has been solved by the ICO learning-based speed adaptation control technique, which can automatically and quickly learn online to perform safe braking through the adaptive weight or gain of the predictive signal. Furthermore, the robustness of the proposed speed adaptation control has been proven by flying the UAV in a variety of situations, such as when confronted with static and dynamic obstacles, and unexpected wind perturbation.

The complexity of our proposed neural control and online learning for speed adaptation control can be determined as follows: (1) the number of neurons in the control network, (2) learning parameters and learning trials (or learning time) required, and (3) input information required for speed adaptation control. The neural control network consisted of only three neurons (i.e., N_1_, N_2_, and R_*m*_; Figure 3). The learning process was based on a simple correlation-based learning rule (Equation 3) with only one learning parameter (i.e., predictive weight). It required a few learning trials (3–4 trials with an average learning time of 464.81 s; Table 1). Additionally, the total learning time of the three control parameters for speed adaptation [i.e., three speed gains (predictive weights) in the x-y direction, z direction when flying upward, and z direction when flying downward] requires approximately 1,493.02 s (or 24.88 min). This remains less than the total optimization time of the MPC (24.48 h) for one speed gain (predictive weights) in the x-y direction (Table 1). It uses minimal information (i.e., only one simple distance sensor) as the input to the control network to predict the system motion dynamics and adapt moving/flying speed of the system. Thus, the complexity of its implementation (see the pseudocode in Section 2.2) and computation (Table 1) is low compared to the MPC.

The speed control function represents the fundamental behavior of a UAV and can be used to support more complex behaviors, such as, obstacle avoidance and exploration. In several state-of-the-art UAV control techniques, such as MPC and learning-based control (e.g., deep learning and RL), speed control is determined as a part of optimizing the control parameters. However, the MPC (Bareiss et al., 2017; Lindqvist et al., 2020; Wang et al., 2021) requires a dynamic model of the UAV, the current state of the UAV, and the surrounding perceived environment to predict the future state and determine the optimal control parameters (as shown in Supplementary Material). These control parameters can be the positions, flying speeds, and acceleration, depending on the system and mission goal. Similarly, in deep learning (Kaufmann et al., 2019; Varshney et al., 2019; Palossi et al., 2022) and RL (Shin et al., 2019; Singla et al., 2019), these parameters are optimized through the learning process, which typically requires a large number of dataset and numerous iterations. In comparison to existing state-of-the-art UAV control techniques (Table 2), our neural predictive control technique does not require a UAV dynamic model, a simulated environment for learning, nor does it require the collection of a training dataset in advance. Apart from a UAV localization system, the control technique uses only a single input from a distance sensor and employs a few learning trials to online learn/optimize control parameter(s) for speed adaptation. The resulting speed adaptation behavior is also robust against static and dynamic obstacles, as well as against wind perturbation. To the best of our knowledge, the achievement realized by simple and data-efficient online learning control has not been demonstrated by others (Table 2). Furthermore, the control technique can be implemented in a modular architecture (Figures 2, 3). The architecture is flexible, with a module that can be added or removed to increase or decrease the ability of the control system to meet task requirements. This architecture also allows the independent development of each module, which reduces the overall complexity of the control system.

**Table 2:** A comparison between our proposed and state-of-the-art methods in terms of system requirement for implementing each method, experimental UAV platform, offline/online learning or optimization process, input information required to perform a task, UAV tasks or behaviors, and environmental uncertainty.

**Method**	**System Requirement**			**UAV platform**		**Learning/** **Optimization**		**Input Informations**	**UAV Behaviors**	**Environmental** **Uncertianty**	
	**UAV Dynamic Model**	**Pre-collection Training Dataset**	**Simulated environment for learning**	**Sim**	**Real**	**Offline**	**Online**			**Wind Perturbation**	**Dynamic Object**
Bareiss et al., 2017^1^	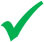	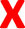	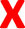	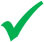	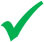	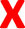	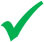	UAV's state and 2D (360°) obstacle distance	Obstacle avoidance	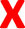	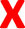
Lindqvist et al., 2020^1^	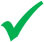	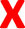	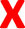	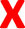	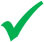	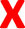	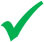	UAV's state and obstacle state	Obstacle avoidance	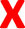	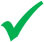
Wang et al., 2021^1^	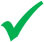	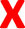	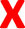	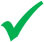	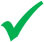	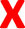	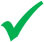	UAV's state	Trajectory tracking	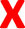	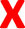
Kaufmann et al., 2019^1, 2^	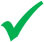	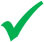	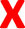	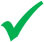	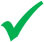	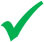	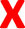	Visual and UAV's state	Trajectory planning and tracking	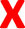	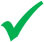
Varshney et al., 2019^1, 2^	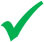	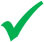	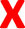	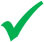	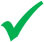	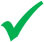	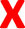	UAV's state	Trajectory tracking	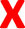	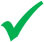
Palossi et al., 2022^2^	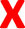	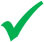	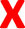	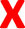	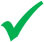	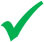	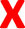	Visual	Object pose estimation and following	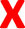	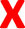
Shin et al., 2019^3^	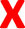	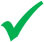	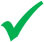	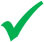	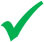	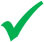	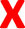	Visual	Obstacle avoidance	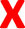	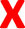
Singla et al., 2019^3^	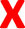	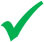	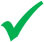	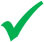	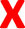	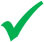	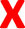	Visual	Obstacle avoidance	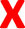	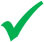
Our NPC	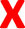	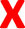	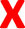	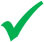	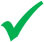	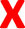	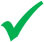	Obstacle distance (from a distance sensor)	Speed adaptation and obstacle avoidance	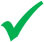	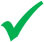

*Note that^1^ is MPC,^2^ is deep learning, and^3^ is RL*.

Although the speed adaptation control was designed to use with different maximum UAV flying speeds, we validated it at a maximum speed of up to 1.5 m/s because of the limitations of flying space and the experimental setting where the UAV was unable to fly at a speed faster than 1.5 m/s for a safety reason. Moreover, when considering the statistical data in Figure 7, we determined that the variance of the approximated predictive weight and braking position of the UAV tends to be high when flying at a fast speed (i.e., 1.5 m/s) compared with other speeds (i.e., 0.5 and 1.0 m/s). This might be because we used the same predictive and reflective ranges for all tested speeds, which might be non-optimal. Additionally, the compact optical distance measurement sensor used in this study was the LiDAR-Lite V3, which is a narrow beam sensor. This might cause inaccurate measurement when the shape and size of the obstacle are not suitable or too small for this measurement technique. In addition, our proposed method still relies on a non-neural system to store or memorize the learned predictive weights at different flying speeds, perform polynomial regression for predictive weight approximation, and select a proper predictive weight (*W*_1_) value based on the current maximum speed. Thus, the remaining issues include further investigation of the system's behavior corresponding to the predictive and reflective ranges, using more accurate sensor systems with automatic sensor range adaptation, broadening its capability to higher flying speeds, and improving its robustness. Furthermore, we will develop different neural modules to replace the existing non-neural system toward autonomous lifelong learning and adaptation. The neural modules include (i) a neural memory network (Tetzlaff et al., 2015; Herpich and Tetzlaff, 2019; Auth et al., 2020) for control parameter memorization, (ii) a hypernetwork for control parameter approximation (von Oswald et al., 2019; Galanti and Wolf, 2020), and (iii) a decision-making network for proper control parameter selection and action planning (Arena et al., 2011).

## 5. Conclusion

In this study, we introduced neural proactive control by combining a simple neural control network and a fast online learning algorithm for the speed adaptation of UAVs. The neural control was based on a three-neuron network, whereas the online learning algorithm was derived from different time scales of earlier (predictive) and later (reflexive) signals with neural correlation-based learning between the two signals. In our setup, these signals were generated from only one input distance sensor signal. The predictive signal was mapped to the long-range detection of a prior occurrence. The reflexive signal was mapped to the short-range detection of a subsequent event. The correlation between these two signals was used to learn the synaptic weight of the predictive signal and determine the optimal weight or speed adaptation gain for each UAV's maximum flying speed. Different optimal weights can be utilized to generate different adaptation rates when flying at different maximum speeds, allowing the UAV to decrease its flying speed and brake at a safe distance from an obstacle. The proposed technique was implemented on different simulated and real UAV systems to demonstrate its applicability to different UAV platforms. Because the neural control technique does not rely on a system dynamic model, it is general and can be directly applied to other UAVs. Finally, we evaluated the performance of the developed method in both environments and compared its performance with those of the reactive control and MPC methods.

The performance of the proposed method was demonstrated by the relationship between the maximum overshoot positions and maximum flying speeds of the UAV when flying using speed adaptation control and conventional reactive control (with and without speed adaptation, respectively). The results showed that, when flying with speed adaptation control, the UAV was able to adapt its speed and brake at a safe distance from the virtual obstacle in horizontal and vertical directions. However, when flying without the speed adaptation method, the UAV was unable to brake at a safe distance. At most speeds, the UAV hit or flew beyond the virtual obstacle line. This implies that, when flying without speed adaptation control, the UAV collides with an obstacle in a real environment. For the performance comparison with the MPC, the results show that both control techniques were capable of controlling the UAV to brake at a desired distance from an obstacle. Nevertheless, the MPC was less effective at high speeds (i.e., 1.5 m/s; Figure 12B). Furthermore, it had more system requirements, such as the dynamic model of the UAV and system states (e.g., distance from obstacle or position, speed, and acceleration), and required high computational effort in the optimization process (Table 1). In contrast, the proposed neural control required only a distance from an obstacle with a few learning trials (less computational effort, Table 1). Moreover, we demonstrated the robustness of the speed adaptation control against wind perturbation and its combination with reactive obstacle avoidance control for UAV navigation in a real environment with obstacles in which the UAV could safely avoid obstacles for all trials.

In the future, we will further improve our system by solving the remaining issues mentioned in the section 4. Furthermore, we will integrate a synaptic scaling mechanism (Tetzlaff et al., 2011; Grinke et al., 2015) into our learning mechanism. The synaptic scaling acts as a depression mechanism and can decrease and stabilize the predictive weight. We will also combine the speed adaptation control with adaptive obstacle avoidance control (Pedersen and Manoonpong, 2018) to expand the capability of the UAV toward a safe and effective navigation system. This will enable the UAV to fly at different speeds and proactively adapt its flying speed with safe and efficient obstacle avoidance. Moreover, the UAV will be able to complete a mission faster and safer than flying at the same speed in all areas. Furthermore, we aim to develop other advanced functions, such as goal-directed navigation, adaptive exploration and transportation, and autonomous landing to achieve fully autonomous UAVs for complex real-world applications.

## Data Availability Statement

The original contributions presented in the study are included in the article/Supplementary Material, further inquiries can be directed to the corresponding author.

## Author Contributions

VJ and PM conceived the research idea. VJ developed and implemented the control methods, analyzed the data, and wrote the manuscript. VJ and KR performed experiments and collected information. PM provided the general direction of the study, supervised the development of the neural control system, helped with data analysis, and revised the manuscript. EE discussed the study and reviewed the manuscript. All authors contributed to the article and approved the submitted version.

## Funding

This study was supported by a startup grant on Bio-Inspired Robotics (PM, project PI) from the Vidyasirimedhi Institute of Science and Technology.

## Conflict of Interest

The authors declare that the research was conducted in the absence of any commercial or financial relationships that could be construed as a potential conflict of interest.

## Publisher's Note

All claims expressed in this article are solely those of the authors and do not necessarily represent those of their affiliated organizations, or those of the publisher, the editors and the reviewers. Any product that may be evaluated in this article, or claim that may be made by its manufacturer, is not guaranteed or endorsed by the publisher.

## Figures and Tables

**Figure A1:**
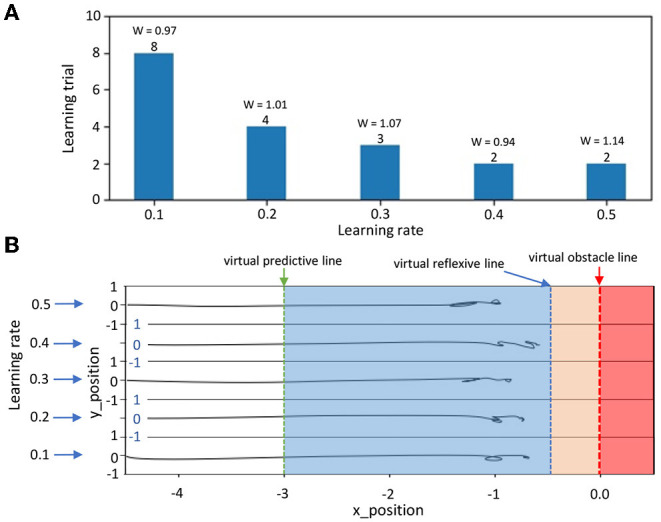
**(A)** Learning trial required and the learned predictive weight for each learning rate. **(B)** Top view position tracking when the UAV flew using the learned predictive weights in **(A)**.

## Footnotes

^1^ https://gitlab.com/BRAIN_Lab/public/neural-control-and-online-learning-for-speed-adaptation.git

^2^The strong stability and fast convergence of the ICO have been analyzed in Porr and Wörgötter (2006).

^3^Note that during the learning process of the real UAV, we used an input signal from a motion capture localization system (OptiTrack) only for safety; see Section 3 for further details.

^4^Note that in the case of using the input signal from OptiTrack, the output of N_1_ can vary between 0.0 (i.e., the distance between the drone and virtual obstacle is greater than 3.0 m) and 1.6 (i.e., the drone flies beyond the virtual obstacle for 1.0 m).

^5^At a fast speed, the UAV may crash the obstacle during the learning process. Thus, to avoid damage, we used the virtual obstacle with the localization system-based input, rather than a real physical obstacle.

^6^Note that, in principle, the control without the speed adaptation is a conventional reactive control method with a predefined sensory feedback gain (i.e., here 1.0), widely used in various UAV behavioral adaptation tasks (Rambabu et al., 2015; Kramer and Kuhnert, 2018). Thus, we basically compare our speed adaptation with the conventional method. In contrast, our control with the speed adaptation uses a proper learned sensory feedback gain with respect to the flying speed.

## Appendix A: The Effect of Different Learning Rates

This section provides an investigation of how the learning rate (μ) affects the system's performance by comparing five different learning rates (i.e., 0.1, 0.2, 0.3, 0.4, and 0.5) under a flying speed of 1.0 m/s. Figure A1 shows that different learning rates resulted in different learning trials. The system can learn quickly (fewer trials) at a high learning rate and vice versa. The learned predictive weights converged to different values at different learning rates. When flying the UAV with different predictive weights, the control system braked the UAV at different distances from the obstacle (Figure A1B). Here, we used a learning rate of 0.3, which allowed for fast learning (three trials) while keeping the UAV at an appropriate distance from the virtual reflexive line (i.e., not too close or not too far from the line compared with the other learning rates). This ensures that the UAV can be at a safe distance when facing external disturbances such as wind perturbation or dynamic obstacles.

## Appendix B: The Result on Speed Adaptation During Learning at Different Speeds

This section provides real-time data of the speed adaptation during online learning. Figure B1 shows a maximum speed of 1.0 m/s. Figure B2 shows a maximum speed of 1.5 m/s.

## Appendix C: The Result Of Speed Adaptation During A Verification Period At A Maximum Speed of 1.25 m/s

This section (Figure C1) provides real-time data on speed adaptation during a verification period at a maximum speed of 1.25 m/s, using the approximated predictive weight of 1.07 (the weight from Figure 7) as the initial predictive weight.
