# Supplementary material for: Neural Control and Online Learning for Speed Adaptation of Unmanned Aerial Vehicles
Source: Front Neural Circuits. 2022 Apr 25;16:839361. doi: 10.3389/fncir.2022.839361 (PMC9082606; doi:10.3389/fncir.2022.839361)
Supplement: Supplementary file 8 [file Supplementary_Material.PDF]

Supplementary Material for "Neural Control and Online Learning for Speed Adaptation of Unmanned Aerial Vehicles"

1 OVERVIEW

This supplementary material contains detailed results and a comparison of our proposed neural control with online learning and model predictive control (MPC) in the Gazebo robot simulation. Both compared control techniques are implemented on a laptop with Intel core i7-9750H 2.6GHz CPU, 8GB RAM, and GeForce GTX 1050 GPU. Therefore, any comparison provided here is based on this computational efficiency. Additionally, the material provides detailed results of the vertical plane experiment to learn the predictive weights for speed adaptation in both the real and simulated UAVs.

2 NEURAL CONTROL AND ONLINE LEARNING IN SIMULATION ENVIRONMENT

Here, we repeated the first experiment described in the main paper in the Gazebo physical simulation environment. An IRIS UAV model in the Gazebo with ardupilot software in the loop (SITL) (Team, 2020) was used as an experimental platform (see Supplementary Fig. S1). We let the UAV online learn the predictive weight of the neural control at different flying speeds using position feedback from the simulation as an input of the neural control. In these experiments, we used a learning rate of 0.1 to allow the system to learn the weight gradually. We flew the UAV at different maximum flying speeds of 0.5, 0.7, 0.9, 1.0, 1.1, 1.3, and 1.5 m/s. The learning results at speeds of 0.5, 1.0, and 1.5 m/s are shown in Supplementary Figs. S2(a), (b), and (c). We also repeated the experiment seven times at each speed to observe the variance of the system. An approximated relationship between predictive weights and maximum flying speeds was calculated using polynomial regression based on the three learned weights (see Supplementary Figs. S2(d)).

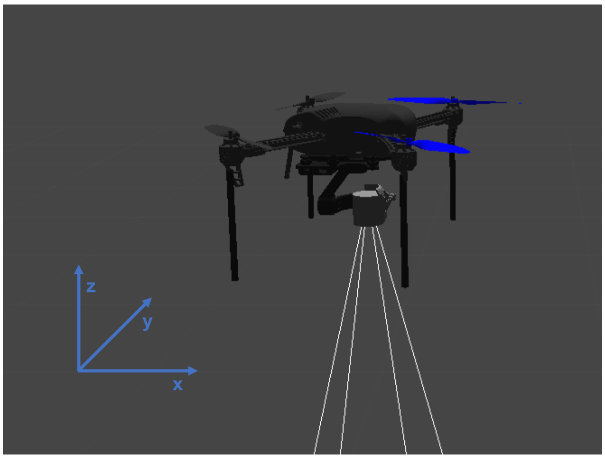

Supplementary Fig. S1. An IRIS UAV model in the Gazebo simulation using ardupilot software in the loop (SITL) as a flight controller.

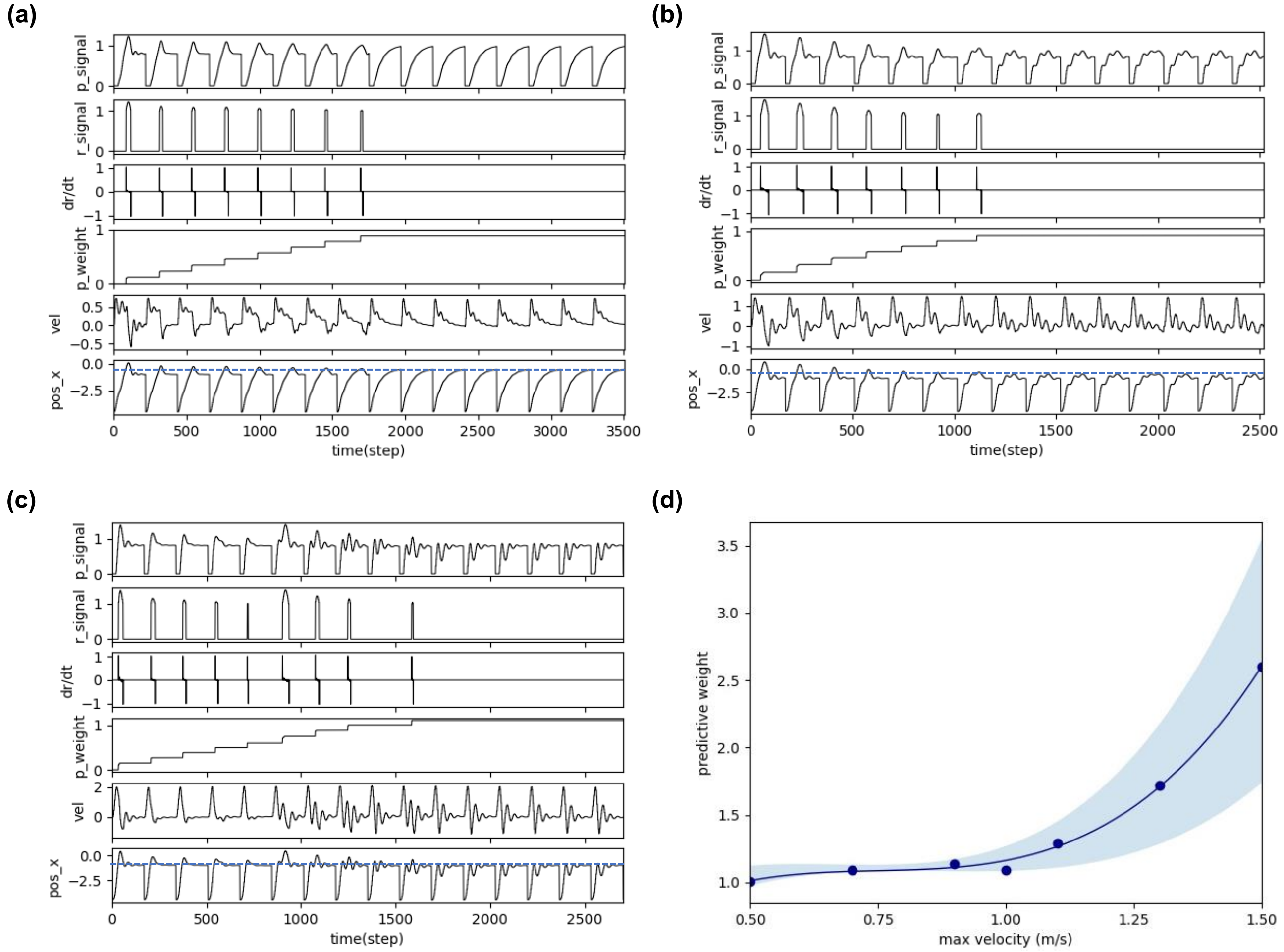

Supplementary Fig. S2. Real-time data of speed adaptation during online learning while flying the UAV forward in the simulated environment at maximum speeds of (a) 0.5 m/s, (b) 1.0 m/s, and (c) 1.5 m/s. (d) The approximated predictive weights based on the learned weights at maximum flying speeds of 0.5, 0.7, 0.9, 1.0, 1.1, 1.3, and 1.5 m/s. Note that p_signal denotes a predictive signal, r_signal denotes a reflexive signal, dr/dt denotes derivative of the reflexive signal, p_weight denotes a predictive weight, vel denotes an actual velocity (x-axis) of the UAV, pos_x denotes a position on the x-axis of the UAV compared to the virtual reflexive line (blue line).

The usability of the approximated weights was verified by flying the UAV at two other different speeds (0.75 and 1.25 m/s) using the initial predictive weights of the corresponding speeds, which are 0.92 and 1.01, respectively (see Supplementary Fig. S2(d)). The results show in Supplementary Fig. S3(a) and (b).

3 MODEL PREDICTIVE CONTROL(MPC)

In this section, we implemented the speed adaptation control using the model predictive control (MPC) (Bareiss et al., 2017; Baca et al., 2018; Lindqvist et al., 2020; Wang et al., 2021), as the main controller of the UAV in the simulated environment. The MPC is an optimization-based control algorithm that generates the optimal control signals by predicting future states of the system in horizon time. It optimizes the control input command to get the lowest cost based on a defined cost function. The implementation detail is described in two subsections. First, system identification and modeling, where we defined parameters and created a dynamics model of the UAV, is given. Second, an explicit MPC (Lee and Chang, 2018; Varshney et al., 2019) for the real-time speed adaptation controller is presented.

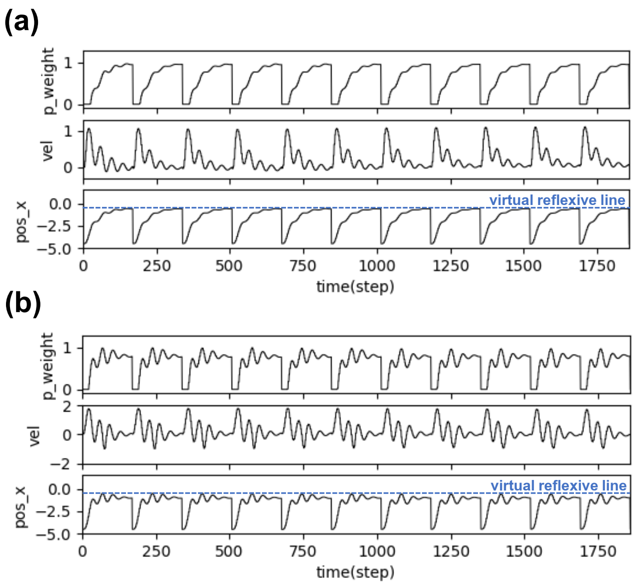

Supplementary Fig. S3. Real-time data when flying the UAV forward in the simulated environment at maximum speeds of (a) 0.75 m/s and (b) 1.25 m/s, using approximated predictive weights of 0.96 and 1.14 (from Supplementary Fig. S2(d)), respectively. Note that p_signal denotes the predictive signal, vel denotes the actual velocity (x-axis) of the UAV, pos_x denotes the position on the x-axis of the UAV compared to the virtual reflexive line (blue line).

3.1 System Identification and Modeling

In horizontal plane experiments, the speed adaptation was simplified by considering only the flying forward of the UAV. Thus, to implement the MPC, we simplified the dynamic model of the UAV by considering the x-axis with respect to the UAV frame. We assumed that the velocity response of the UAV in the x-axis was the second-order response. Therefore the approximated dynamic (state-space) model of the UAV system was derived from the standard form of the second-order closed-loop transfer function as below:

$$2^{nd}\, order\, T.F. = \frac{b^2}{S^2 + 2aS + b^2}, \tag{S1}$$

where S is the complex frequency domain parameter in Laplace transform. b is the undamped natural frequency of the system, a is the undamped natural frequency multiply with the damping ratio.

According to the second-order transfer function described in Eq. S1, an approximated dynamic (state-space) model of the system can be written as follows:

$$\dot{\mathbf{x}} = \mathbf{A}\mathbf{x} + \mathbf{B}u, \tag{S2}$$

$$\begin{bmatrix} \dot{x} \\ \ddot{x} \\ \dddot{x} \end{bmatrix} = \begin{bmatrix} 0 & 1 & 0 \\ 0 & 0 & 1 \\ 0 & -\alpha & -\beta \end{bmatrix} \begin{bmatrix} x \\ \dot{x} \\ \ddot{x} \end{bmatrix} + \begin{bmatrix} 0 \\ 0 \\ -\alpha \end{bmatrix} u, \tag{S3}$$

where $\dot{\mathbf{x}}$ is the derivative of the state vector (velocity ($\dot{x}$), acceleration ($\ddot{x}$), jerk ($\dddot{x}$)), $\mathbf{x}$ is the state vector (position (x), velocity ($\dot{x}$), acceleration ($\ddot{x}$)), $\mathbf{A}$ is the system matrix, $\mathbf{B}$ is the input matrix, α is equal to b^2, β is equal to $2a$, and u is the input command (velocity command in the x-axis).

According to the derived system identification, we flew the UAV with the step input command (flew the UAV at speed 1.0 and -1.0 m/s alternately for a certain period of time), then recorded all states of the UAV, control input (velocity command), and running times. From the recorded data, we approximated parameters α and β (see Eq. S3) of the dynamic model by using the BFGS optimization method (Virtanen et al., 2020) and applied the sum of square error as the cost function. The optimization algorithm is shown in Supplementary Algorithm 1. By doing so, the approximated parameters were α = 2.05038782, β = 1.3975637. Furthermore, we also compared the velocity response of the system using the approximated dynamic model and the actual recorded data of the reference system (Supplementary Fig. S4).

Supplementary Algorithm 1 Parameters Approximation

$dataset \leftarrow load\_reference\_dataset(v_{act}, u_{ref}, t)$
$mod\_param \leftarrow [1,\ 1]$ ▷ initial dynamic model parameters
$dynamic\_model \leftarrow create\_dynamic\_model(mod\_param)$
function PARAM_EST($c\_input, uav\_state, mod\_param, mpc\_param, x_{ref}$)
  $cost,\ v\_est \leftarrow 0$ ▷ initial cost value and estimate velocity
  $N \leftarrow dataset\ length$
  for i **in** $dataset\ length$ **do**
    $t_i,\ u_i,\ v_i \leftarrow dataset.t[i],\ dataset.u_{ref}[i],\ dataset.v_{act}[i]$
    $v\_est_i \leftarrow dynamic\_model(u_i, t_i, v\_est_{i-1})$ ▷ scipy solve an initial value problem for a system of ODEs
    $cost \leftarrow cost + \frac{(v_i - v\_est_i)^2}{N}$ ▷ calculate cost value (sum of square error)
  return $cost$
$output \leftarrow minimize($PARAM_EST$, mod_param, method = (BFGS))$ ▷ scipy BFGS optimization method

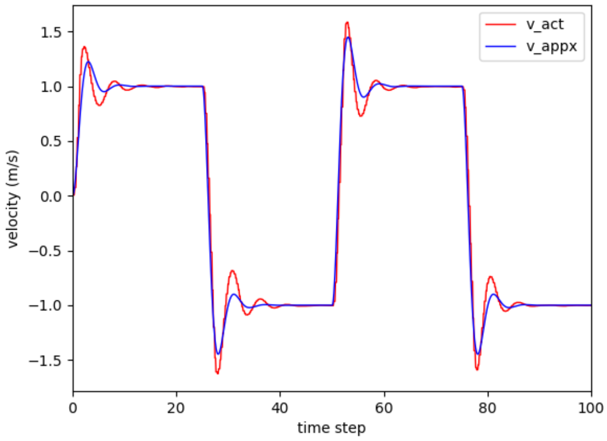

Supplementary Fig. S4. The performance comparison of the velocity step response between the actual (`v_act`) and approximated (`v_appx`) systems.

3.2 Explicit MPC for Speed Adaptation

Since the online computational speed for optimizing and generating a control command of the MPC required among calculation time and was not suitable for the UAV system, we applied an explicit MPC technique instead. The explicit MPC is the implementation technique of the MPC which requires less online computation effort by using pre-offline computation to optimize the control command for all possible

operating states. The computed result can be stored in a form of a table for looking up when performing tasks or in a form of a neural network that is trained using the computed result as the training data set . In this experiment, we used a table for implementing the explicit MPC as the speed adaptation controller of the UAV. To execute this, the first step was the pre-offline optimization to find the optimal control command for every possible states of the UAV. Therefore, we designed the cost function as shown in Eq. S4. The optimization process was executed using Trust-Region Constrained optimization method (Virtanen et al., 2020) to minimize the cost function. The MPC (pre-offline) optimization diagram and algorithm are shown in Supplementary Fig. S5 and Supplementary Algorithm 2. By doing so, we obtained the optimized result for all possible states of the UAV and their optimal control input command.

$$\min_{u} J(x,u) = \sum_{i=1}^{N} \left(w_x(x_{ref} - x_i)^2 + w_u(u_i - u_{i-1})^2 + w_{os}\left(\frac{(x_{ref} - x_i) - |x_{ref} - x_i|}{2}\right)^2 \right), \quad \text{(S4)}$$

where J is the cost function. x is the position of the UAV. u is the control input. w_x is the weighting coefficient reflecting the relative importance of the position error which is set to 5. w_u is the weighting coefficient reflecting the relative importance of the derivative of the control input which is set to 10. w_{os} is the weighting coefficient reflecting the relative importance of the derivative of the overshoot position error which is set to 100. N is the control horizon.

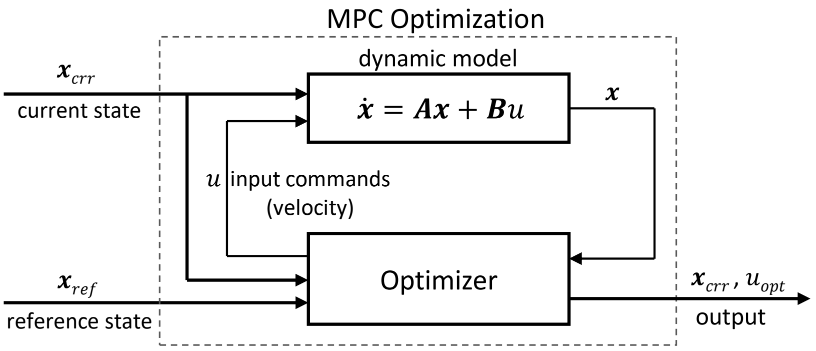

Supplementary Fig. S5. Diagram of the MPC optimization.

The implementation diagram of the speed adaptation using the neural control and the explicit MPC as the main controller are presented in Supplementary Fig. S6(a) and S6(b), respectively. From the optimized result getting in the previous step, we used it as the speed adaptation controller by creating the closed-loop speed adaptation control as shown in Supplementary Fig. S6(b). The dashed lines show the pre-offline computed to optimize the control commands for all states of the UAV which were stored in a form of a matching table between each state of the UAV and its optimal control input command. The solid lines show the closed-loop control using currently state of the UAV as an input to look up its optimal control command from the table and then feed into the UAV to perform speed adaptation. Supplementary Fig. S7 shows the comparative performance graphs of the UAV flying at five different maximum speeds (0.5, 0.75, 1.0, 1.25, and 1.5 m/s) when performing speed adaptation using our neural control and the explicit MPC.

Supplementary Algorithm 2 MPC Optimization

```
function COST_CAL(c_input, uav_state, mod_param, mpc_param, x_ref)
    dynamic_model ← create_dynamic_model(mod_param)
    control_horizon ← mpc_param[0]
    control_period ← mpc_param[1]
    system_period ← mpc_param[2]
    c_range ← (0, 1 ∗ control_period, 2 ∗ control_period, . . . , control_horizon ∗ control_period)
    c_input_appx ← f = approx(c_input, c_range)    ▷ PCHIP 1-D monotonic cubic interpolation
    s_range ← (0, 1 ∗ system_period, 2 ∗ system_period, . . . , control_horizon∗, control_period)
    state_{i−1} ← uav_state    ▷ initial UAV's state
    u_{i−1} ← 0    ▷ initial previous control input value
    sum_cost ← 0    ▷ initial cost value
    for i in s_range do
        t_i ← s_range[i]    ▷ current time (t)
        u_i ← c_input_appx[t]    ▷ control input value at time t
        state_i ← dynamic_model(u_i, t_i, state_{i−1})    ▷ scipy solve an initial value problem for a system of ODEs
        x_i ← state_i.x    ▷ get new UAV's position
        sum_cost ← sum_cost + cost_function(x_ref, x_i, u_i, u_{i−1})    ▷ calculate cost value
    return sum_cost
function MPC_SOLVER(uav_state, c_input_limit, mod_param, mpc_param, x_ref)
    control_horizon ← mpc_param[0]
    c_input ← [0] ∗ control_horizon    ▷ initial a control input a matrix
    u_optimal ← minimize(COST_CAL, c_input, args = (uav_state, mod_param, mpc_param, x_ref), bounds = (−c_input_limit, c_input_limit), method = (trust − constr))    ▷ scipy Trust-Region Constrained optimization
    return uav_state, u_optimal
mod_param    ▷ define model parameters (using estimated parameters)
mpc_param ← [control_horizon, control_period, system_period]    ▷ define mpc parameters to the system
pos_range, vel_range, acc_range    ▷ define possible UAV's state
c_input_limit    ▷ define control input (velocity) limit
x_ref    ▷ define reference position
for pos in pos_range do
    for vel in vel_range do
        for acc in acc_range do
            uav_state ← pos, vel, acc
            output ← MPC_SOLVER(uav_state, c_input_limit, mod_param, mpc_param, x_ref)
```

4 NEURAL CONTROL AND ONLINE LEARNING IN VERTICAL PLANE

Here, we performed a similar experiment to learn the weights of the speed control parameter (predictive weights) in the vertical plane when the UAV flew upward and downward for both in real and simulated environments. The experimental setup is shown in Supplementary Fig. S8. In this experiment, we reduced the predictive range and the free space between the starting point to the virtual predictive line by half compared to the experimental setup in the horizontal plane (see Fig. 5 in the main paper) since the experimental space in the real environment was limited. Additionally, we set the initial predictive weight to 0.5 in order to decrease the overshoot distance of the UAV for a safety reason. Herein, we first started the experiment in a real environment using a learning rate of 0.3. The results in Supplementary Fig. S9(a), (b), and (c) show real-time data during online learning while flying the UAV upward at maximum speeds of 0.5, 1.0, and 1.5 m/s, respectively. The approximated predictive weights with the corresponding maximum flying speeds of the UAV when flying upward are shown in Supplementary Fig. S9(d). The

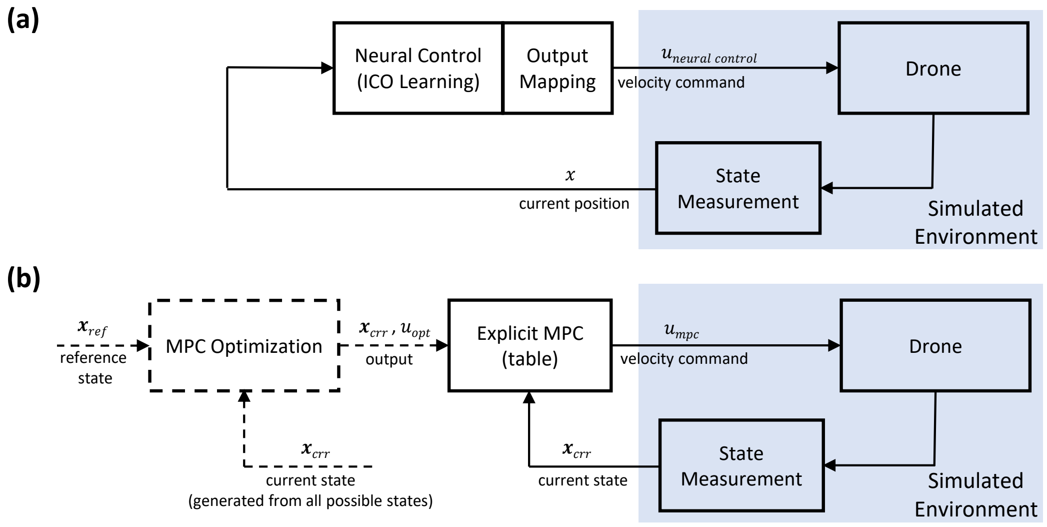

Supplementary Fig. S6. (a) Closed-loop speed adaptation control using the neural control as the main controller. (b) Closed-loop speed adaptation control using the explicit MPC as the main controller. Solid lines show the signal flow in real time, while dash lines show the pre-offline calculation of the MPC optimization.

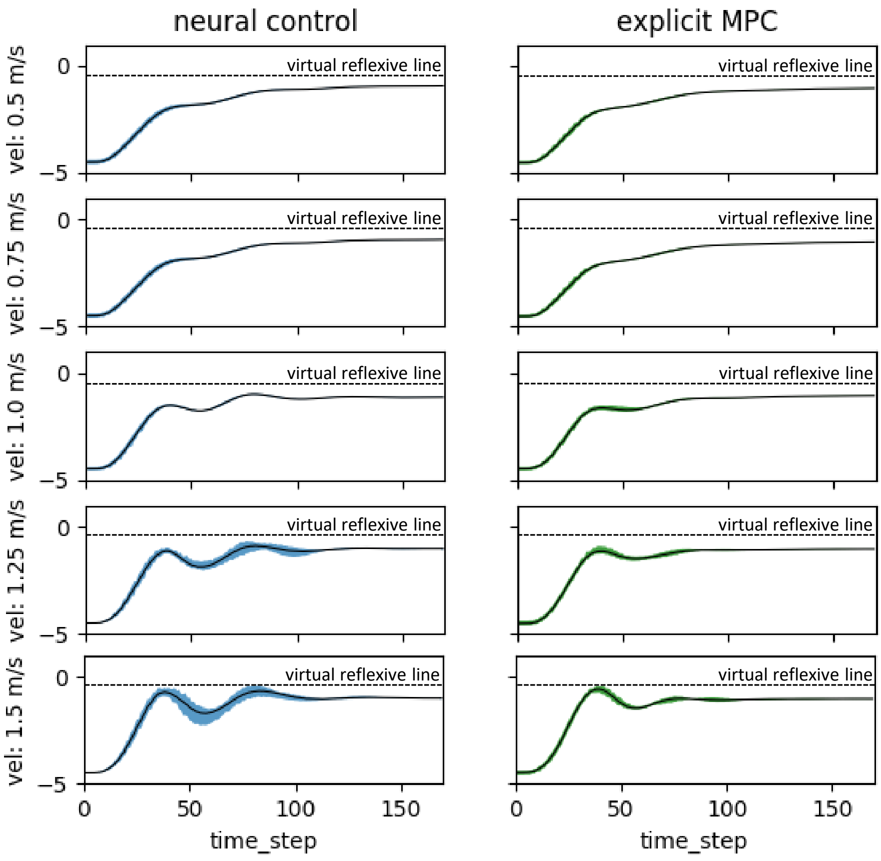

Supplementary Fig. S7. Performance comparison of the UAV, when flying at five different maximum speeds (0.5, 0.75, 1.0, 1.25, and 1.5 m/s) using our neural control and the explicit MPC.

results of flying the UAV downward are shown in Supplementary Fig. S10(a) and (b) for maximum speeds of 0.5 and 1.0 m/s, respectively. The approximated predictive weights when the UAV flying downward are shown in Supplementary Fig. S10(c).

We also verified the usability of the approximated weights by flying the UAV upward at other different speeds (i.e., 0.75 and 1.25 m/s) and flying the UAV downward at another different speed (i.e., 0.75 m/s). We used the initial predictive weights of the corresponding speeds, which are 1.15, 1.18, and 1.15, respectively (see Supplementary Fig. S9(d) and S10(c)). The results show in Supplementary Fig. S11(a), (b), and (c). Furthermore, we repeated these experiments in the simulated environment using a learning rate of 0.1. The results are shown in Supplementary Fig. S12, S13, and S14.

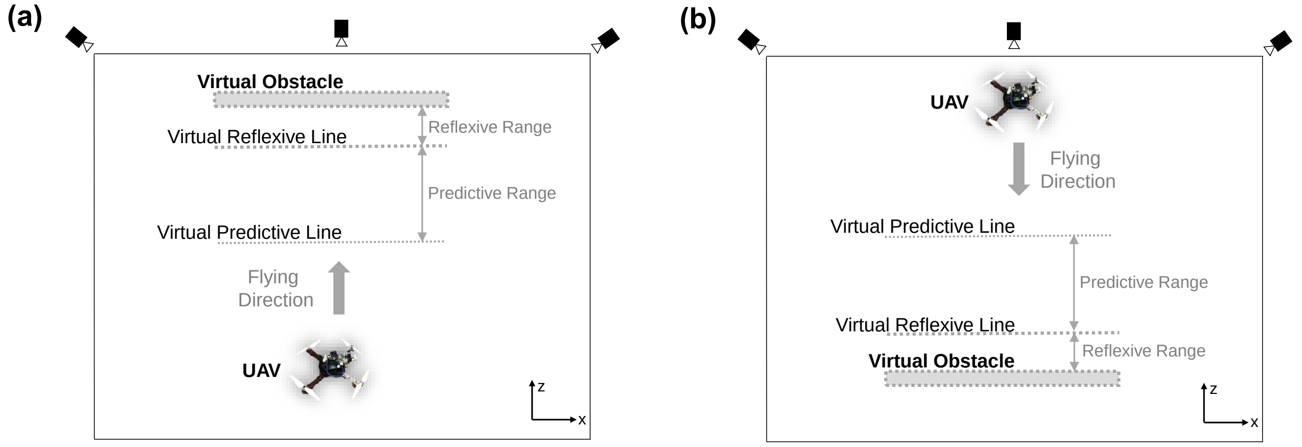

Supplementary Fig. S8. Vertical plane experimental setup for neural control with online learning of a UAV which shows a side view of the flying environment and also predictive and reflexive ranges with respect to the virtual obstacle. (a) Experimental setup for the UAV to fly upward, in this setup, the virtual obstacle was placed at position z = 3.0, with the virtual predictive line and virtual reflexive line located at z = 2.5 m and 1.25 m, respectively. In other words, the predictive range was 1.25 m and the reflexive range was 0.5 m. The UAV started flying at position z = 0.5 m. (b) Experimental setup for the UAV to fly downward, in this setup, the virtual obstacle was placed at position z = 0.5, with the virtual predictive line and virtual reflexive line located at z = 1.0 m and 2.25 m, respectively. In other words, the predictive range was 1.25 m and the reflexive range was 0.5 m. The UAV started flying at position z = 3.0 m.

REFERENCES

Baca, T., Hert, D., Loianno, G., Saska, M., and Kumar, V. (2018). Model predictive trajectory tracking and collision avoidance for reliable outdoor deployment of unmanned aerial vehicles. In *2018 IEEE/RSJ International Conference on Intelligent Robotics and Systems (IROS)*. 6753–6760

Bareiss, D., Bourne, J. R., and Leang, K. K. (2017). On-board model-based automatic collision avoidance: application in remotely-piloted unmanned aerial vehicles. *Auton Robot* 41, 1539–1554. doi:10.1007/s10514-017-9614-4

Lee, J. and Chang, H.-J. (2018). Analysis of explicit model predictive control for path-following control. *PLoS ONE* 13. doi:https://doi.org/10.1371/journal.pone.0194110

Lindqvist, B., Mansouri, S. S., Agha-mohammadi, A., and Nikolakopoulos, G. (2020). Nonlinear mpc for collision avoidance and control of uavs with dynamic obstacles. *IEEE Robotics and Automation Letters* 5, 6001–6008. doi:10.1109/LRA.2020.3010730

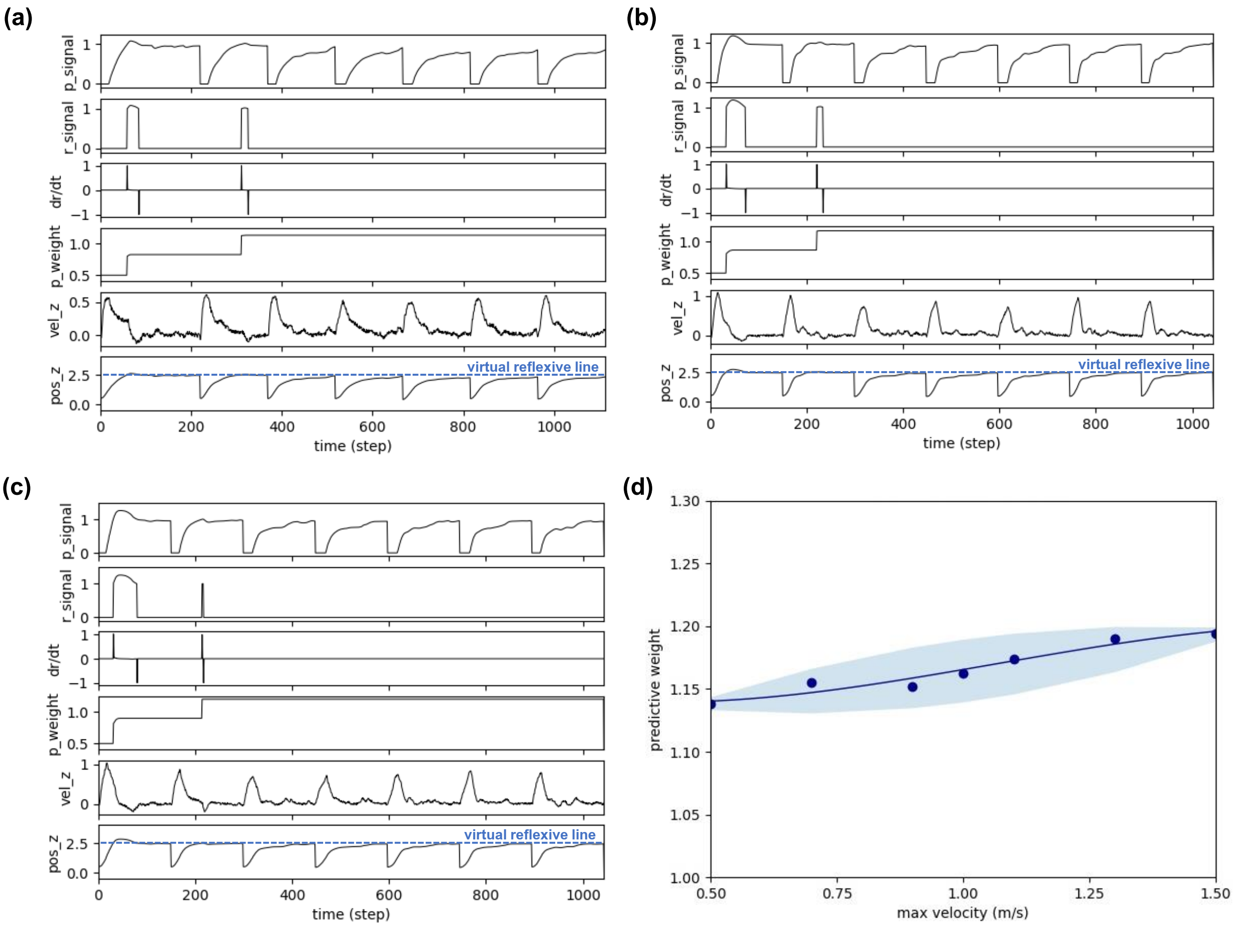

Supplementary Fig. S9. Real-time data of speed adaptation during online learning while flying the real UAV upward at maximum speeds of (a) 0.5 m/s, (b) 1.0 m/s, and (c) 1.5 m/s. (d) The approximated predictive weights based on the learned weights at the maximum flying speeds of 0.5, 0.7, 0.9, 1.0, 1.1, 1.3, and 1.5 m/s. Note that p_signal denotes the predictive signal, r_signal denotes the reflexive signal, dr/dt denotes derivative of the reflexive signal, p_weight denotes the predictive weight, vel_z denotes the actual velocity (z-axis) of the UAV, pos_z denotes the position on the z-axis of the UAV compared to the virtual reflexive line (blue line). A video of this experiment can be viewed on `www.manoonpong.com/DSA/video4.mp4`

[Dataset] Team, A. D. (2020). Using gazebo simulator with ardupilot sitl

Varshney, P., Nagar, G., and Saha, I. (2019). Deepcontrol: Energy-efficient control of a quadrotor using a deep neural network. In *2019 IEEE/RSJ International Conference on Intelligent Robots and Systems (IROS)*. 43–50. doi:10.1109/IROS40897.2019.8968236

Virtanen, P., Gommers, R., Oliphant, T., and et al. (2020). Scipy 1.0: fundamental algorithms for scientific computing in python. *Nat Methods* 17, 261–272

Wang, D., Pan, Q., Shi, Y., Hu, J., and Zhao, C. (2021). Efficient nonlinear model predictive control for quadrotor trajectory tracking: Algorithms and experiment. *IEEE Transactions on Cybernetics* , 1–12doi:10.1109/TCYB.2020.3043361

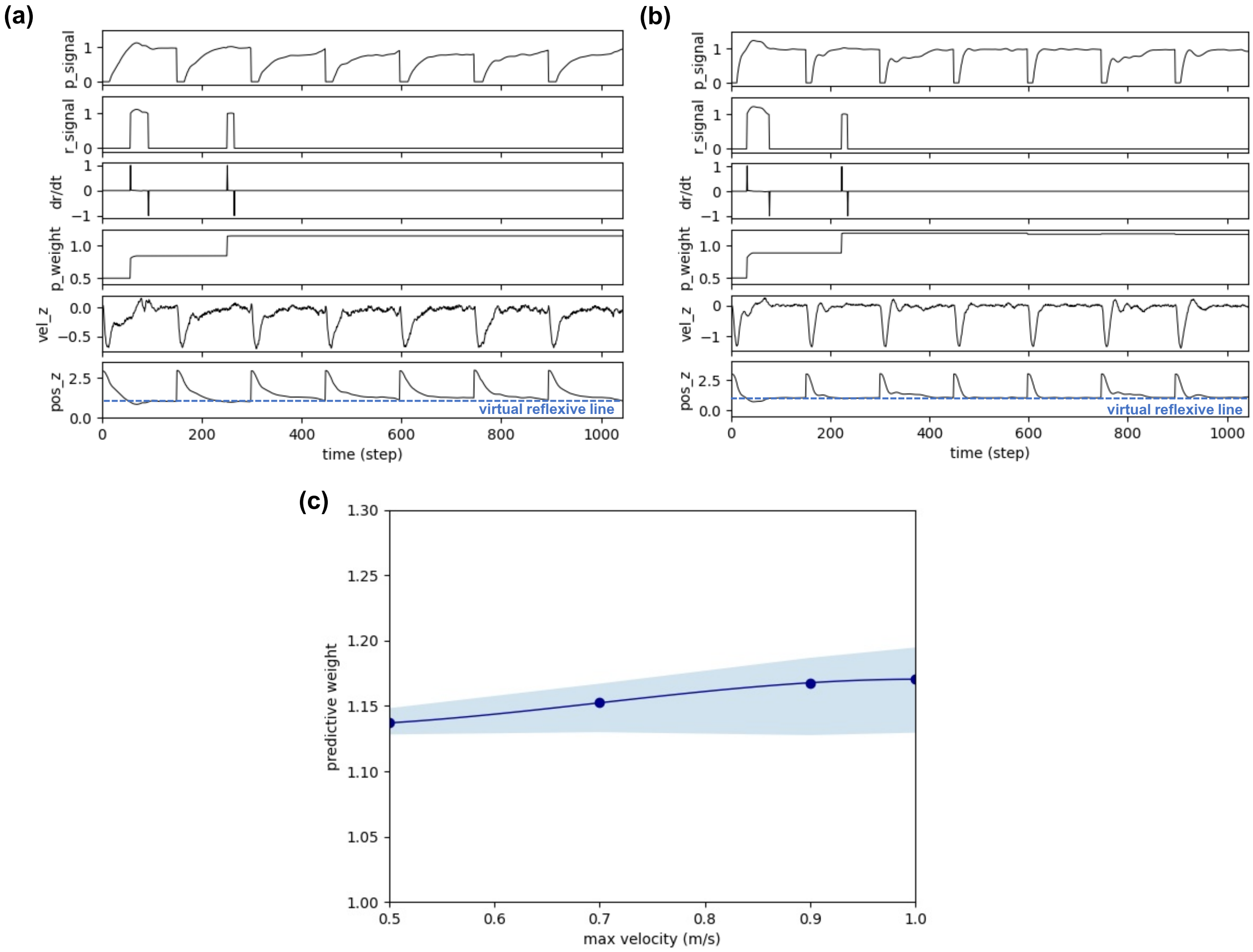

Supplementary Fig. S10. Real-time data of speed adaptation during online learning while flying the real UAV downward at maximum speeds of (a) 0.5 m/s and (b) 1.0 m/s. (c) The approximated predictive weights based on the learned weights at the maximum flying speeds of 0.5, 0.7, 0.9, and 1.0 m/s. Note that p_signal denotes the predictive signal, r_signal denotes the reflexive signal, dr/dt denotes derivative of the reflexive signal, p_weight denotes the predictive weight, vel_z denotes the actual velocity (z-axis) of the UAV, pos_z denotes the position on the z-axis of the UAV compared to the virtual reflexive line (blue line). A video of this experiment can be viewed on `www.manoonpong.com/DSA/video4.mp4`

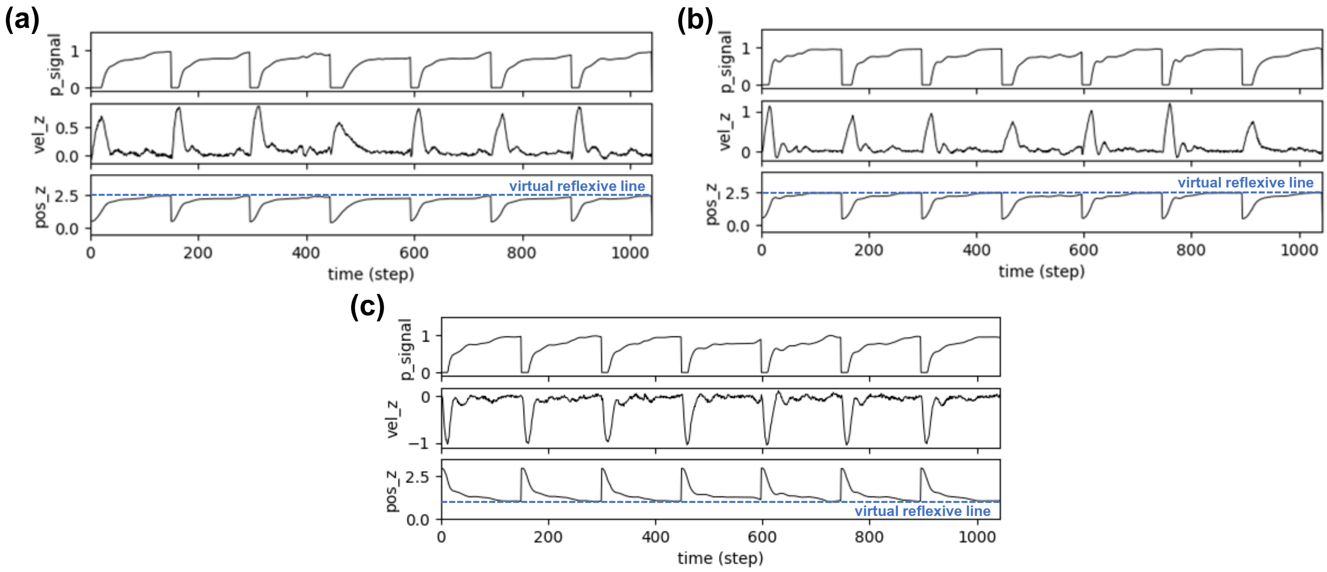

Supplementary Fig. S11. Real-time data when flying the real UAV upward at maximum speeds of (a) 0.75 m/s and (b) 1.25 m/s. (c) Flying the UAV downward at a maximum speed of 0.75 m/s. The used predictive weights were 1.15, 1.18, and 1.15 (from Supplementary Fig. S9(d) and S10(c)), respectively. Note that p_signal denotes the predictive signal, vel_z denotes the actual velocity (z-axis) of the UAV, pos_z denotes the position on the z-axis of the UAV compared to the virtual reflexive line (blue line).

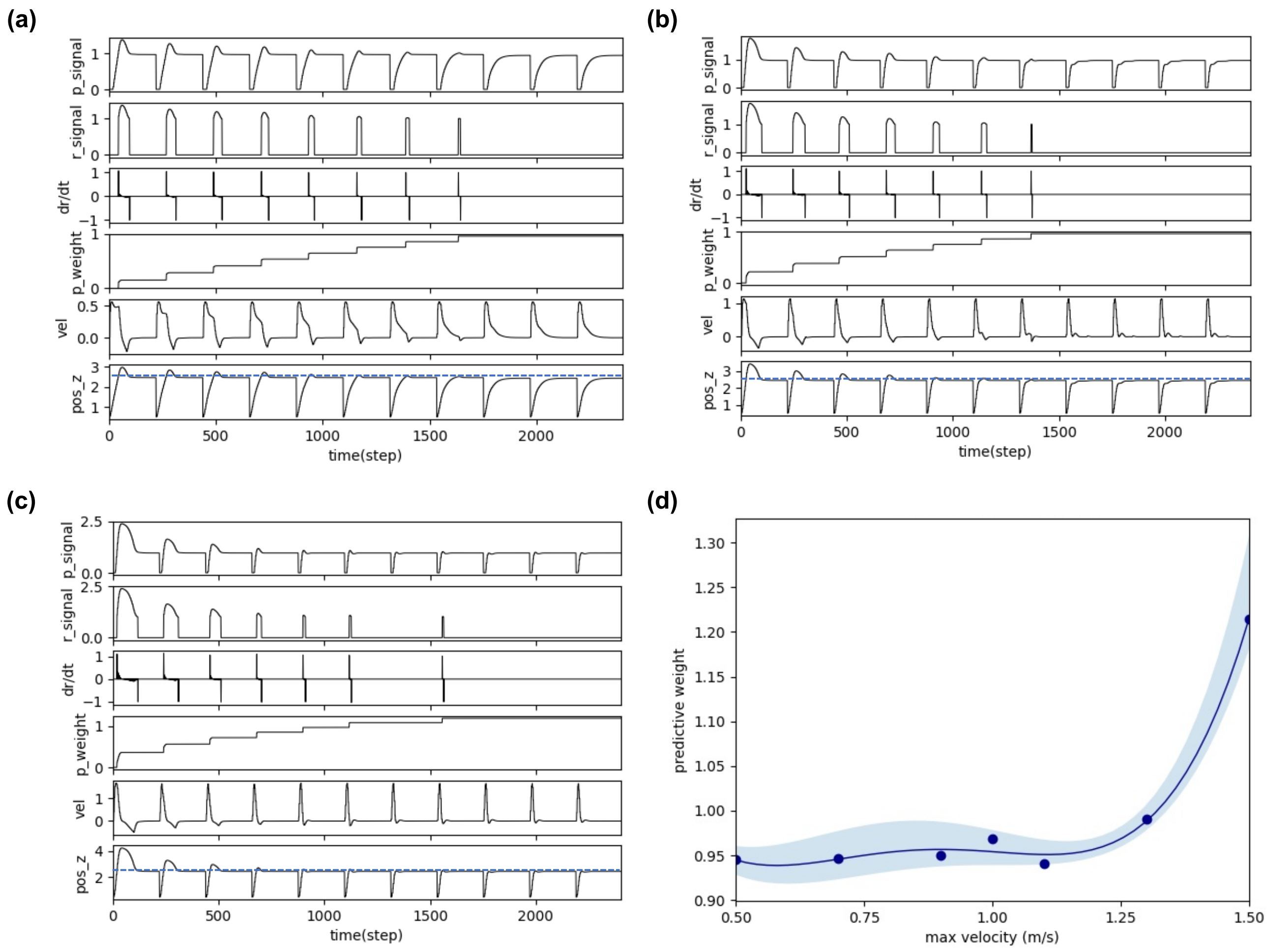

Supplementary Fig. S12. Real-time data of speed adaptation during online learning while flying the simulated UAV upward at maximum speeds of (a) 0.5 m/s, (b) 1.0 m/s, and (c) 1.5 m/s. (d) The approximated predictive weights based on the learned weights at the maximum flying speeds of 0.5, 0.7, 0.9, 1.0, 1.1, 1.3, and 1.5 m/s. Note that p_signal denotes the predictive signal, r_signal denotes the reflexive signal, dr/dt denotes derivative of the reflexive signal, p_weight denotes the predictive weight, vel denotes the actual velocity (z-axis) of the UAV, pos_z denotes the position on the z-axis of the UAV compared to the virtual reflexive line (blue line).

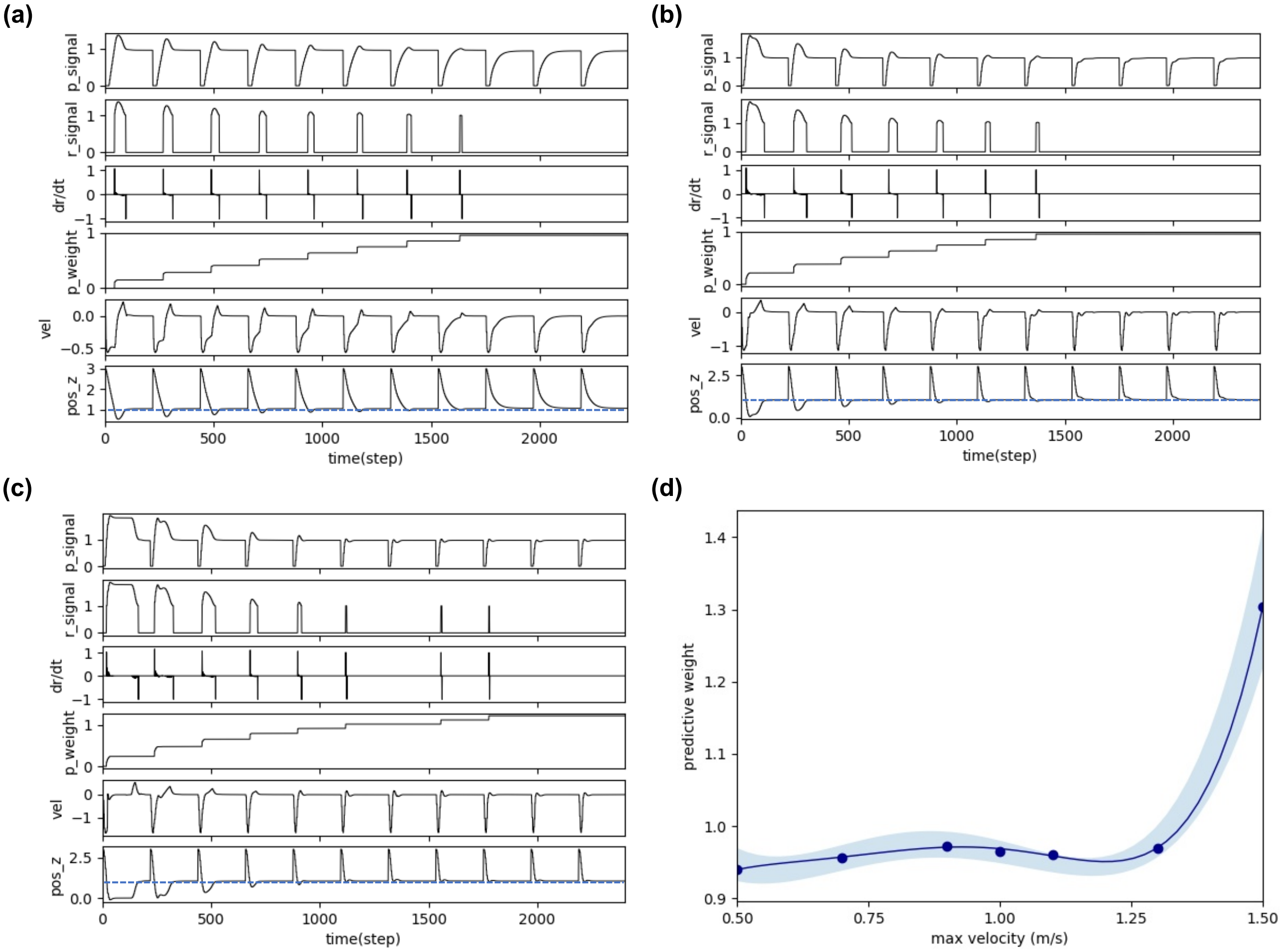

Supplementary Fig. S13. Real-time data of speed adaptation during online learning while flying the simulated UAV downward at maximum speeds of (a) 0.5 m/s, (b) 1.0 m/s, and (c) 1.5 m/s. (d) The approximated predictive weights based on the learned weights at the maximum flying speeds of 0.5, 0.7, 0.9, 1.0, 1.1, 1.3, and 1.5 m/s. Note that p_signal denotes the predictive signal, r_signal denotes the reflexive signal, dr/dt denotes derivative of the reflexive signal, p_weight denotes the predictive weight, vel denotes the actual velocity (z-axis) of the UAV, pos_z denotes the position on the z-axis of the UAV compared to the virtual reflexive line (blue line).

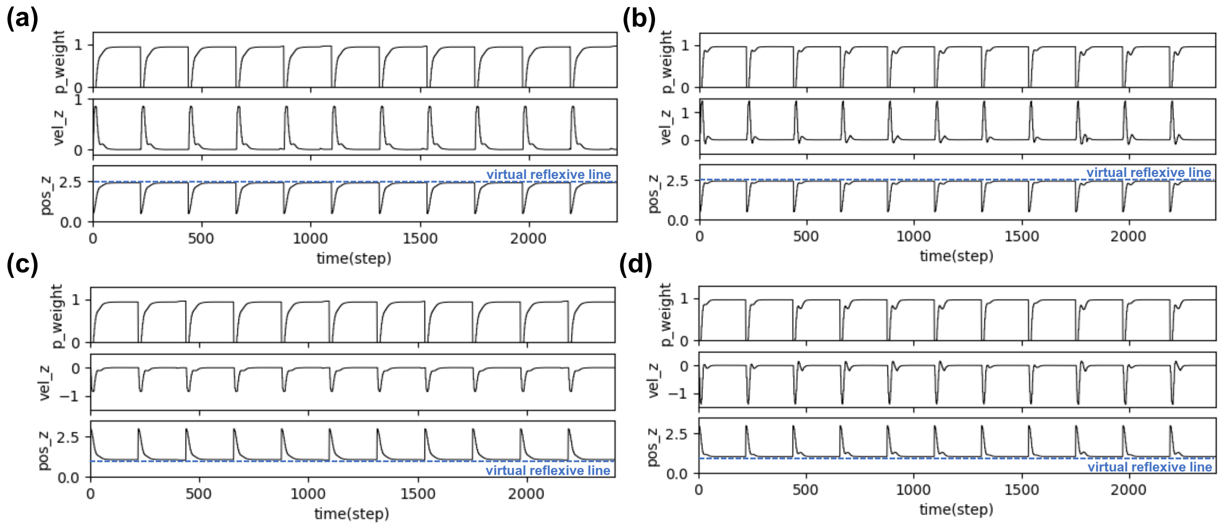

Supplementary Fig. S14. Real-time data when flying the simulated UAV upward at maximum speeds of (a) 0.75 m/s and (b) 1.25 m/s. (c) Flying the UAV downward at maximum speeds of 0.75 m/s and (d) 1.25 m/s. The used predictive weights were 0.96, 0.98, 0.95, and 0.97 (from Supplementary Fig. S12(d) and S13(d)), respectively. Note that p_signal denotes the predictive signal, vel_z denotes the actual velocity (z-axis) of the UAV, pos_z denotes the position on the z-axis of the UAV compared to the virtual reflexive line (blue line).
